# Modeling ultrafast laser excitation of fused silica with a hybrid Lorentz–Drude dielectric response and density-dependent two-temperature model

**DOI:** 10.1038/s41598-026-63133-7

**Published:** 2026-07-27

**Authors:** Daniel Metzner, Philipp Rebentrost, Manuel Pfeiffer, Peter Lickschat, Jonas Opitz, Steffen Weißmantel

**Affiliations:** Laserinstitut Hochschule Mittweida, University of Applied Sciences Mittweida, Mittweida, 09648 Germany

**Keywords:** Materials science, Optics and photonics, Physics

## Abstract

Ultrashort laser excitation of dielectric materials is governed by a complex interplay between nonlinear photoexcitation, transient optical response, and energy transfer to the lattice. A consistent physical description of these processes across different pulse durations remains challenging, as purely free-electron-based optical models and classical thermal approaches do not adequately capture the strongly dynamic and non-equilibrium nature of the excited electron system. In this work, a coupled modeling framework is developed for amorphous fused silica (SiO_2_) that directly links the transient optical response to a density-dependent two-temperature description of the subsequent thermal evolution. The optical properties are described using a hybrid Lorentz–Drude formalism, enabling a consistent representation of bound, localized, and free electronic states during excitation. The resulting energy deposition is coupled to a modified thermal model incorporating electron-density-dependent material parameters derived from first-principles calculations. The model is evaluated by comparison with experimentally measured ablation geometries and single-pulse ablation thresholds for pulse durations in the femtosecond and picosecond range. The threshold analysis indicates that experimentally measurable material removal can occur below the model-internal phase-explosion criterion, particularly for longer pulse durations. In the femtosecond regime, both ablation depth and diameter are reproduced within a limited deviation range. In the picosecond regime, the ablation depth remains consistent with experimental observations, while systematic deviations in the lateral extent become apparent. At longer pulse durations, the experimentally observed structures are increasingly influenced by melt-mediated material redistribution, which is not captured by the present model and leads to pronounced deviations in the ablation geometry. These results demonstrate that the proposed framework provides an effective physical description of ultrafast laser–matter interaction in non-equilibrium and transition regimes, while also defining its limitations in thermally dominated regimes where hydrodynamic effects become significant.

## Introduction

Ultrashort pulsed laser irradiation of dielectric materials such as fused silica enables highly precise micro- and nanostructuring based on nonlinear photoexcitation processes. In contrast to linear absorption, electrons can be excited across the wide band gap via multiphoton and tunneling ionization, followed by avalanche ionization, resulting in a rapid increase of the transient electron population within the conduction band^1–3^. The resulting evolution of the electronic system governs the transient optical properties of the material and thereby directly determines the spatial and temporal energy deposition during laser irradiation.

In the majority of theoretical and experimental studies, the excited electron ensemble is treated as a nearly free electron plasma, and the transient optical response is described using Drude-type models^3,4^. This approach has been widely adopted, in particular because time-resolved reflectivity measurements can often be reproduced within this framework. However, recent spectroscopic pump–probe ellipsometry studies have demonstrated that this description is not sufficient to capture the full transient optical response of fused silica. While Drude-based models can reproduce the transient reflectance and extinction coefficient, they fail to accurately describe the transient refractive index on ultrafast timescales. Instead, the experimental data indicate that the excited electrons cannot be considered as a nearly free electron plasma during the early stages of excitation, but exhibit characteristics of localized, bound-like states^5^. At the same time, a transition towards more free-electron-like behavior at later stages cannot be excluded, indicating that neither purely Drude-based nor purely Lorentz-based descriptions are sufficient over the full interaction regime.

Based on these optical descriptions, a large class of models has been developed to describe laser-induced damage and ablation in dielectrics. Many of these approaches rely on rate-equation frameworks, in which the evolution of the free electron density is used as a central quantity to determine energy absorption and subsequent material modification^2,6^. In this context, the onset of damage or ablation is frequently associated with a critical free electron density, corresponding to a transition to a plasma-like state^6,7^. Alternative approaches employ temperature-based criteria, linking material modification to the attainment of melting or vaporization thresholds, or use simplified energetic considerations based on deposited energy densities^6^. While these approaches have been successfully applied to estimate damage thresholds and ablation depths, they depend strongly on the underlying assumptions regarding the transient optical response and the chosen damage criterion, and do not provide a universally consistent physical description across different excitation regimes.

The coupling of the excited electron system to the lattice is commonly described using the two-temperature model (TTM), which treats electrons and lattice as separate subsystems with distinct temperatures and subsystem-specific material parameters. Originally developed for metals, the classical TTM assumes a high and quasi-constant electron density and employs material parameters such as the electron heat capacity and the electron–phonon coupling factor that are typically derived under these conditions^8^. For wide-band-gap dielectrics such as fused silica, however, the electron density evolves dynamically over several orders of magnitude during laser excitation. In the early stages, the electron density remains comparatively low and evolves dynamically over several orders of magnitude, leading to strongly density-dependent thermodynamic properties of the electron system. In particular, the electron heat capacity becomes strongly dependent on the transient electron density, which is not captured by classical parameterizations. As a consequence, the direct application of classical TTM formulations can lead to inconsistencies in the predicted electron temperature and the energy transfer to the lattice. These limitations have been explicitly recognized in previous modeling approaches, where key quantities such as the electron–phonon coupling factor and the energy transfer terms remain insufficiently described for dynamically evolving electron densities^6^. Recent first-principles calculations^9^ have demonstrated that both the electron heat capacity and the electron–phonon coupling in fused silica strongly depend on the excitation level, further emphasizing the need for an electron density-dependent description of the thermal response.

Beyond these approaches, hydrodynamic, thermo-mechanical, and molecular dynamics simulations have been employed to investigate the subsequent material response following energy deposition. These models provide detailed insights into processes such as stress generation, shock-wave propagation, cavity formation, and atomistic structural rearrangements^10–12^. However, they are typically applied to localized interaction volumes or confined modification regimes and often rely on simplified or externally prescribed energy deposition profiles. As a result, while these approaches are well suited to resolve local material dynamics, they are generally not designed to directly predict complete single-ablation geometries over the full lateral interaction zone extending over several tens of micrometers.

Taken together, these considerations reveal a fundamental inconsistency in the current theoretical description of ultrafast laser excitation and subsequent material response in fused silica. On the one hand, the transient optical response of amorphous fused silica under ultrafast excitation cannot be consistently described by purely free-electron-based models, particularly in the early stages where localized electronic states dominate^5^. On the other hand, commonly used thermal models do not adequately account for the strongly dynamic and non-equilibrium nature of the electron system in dielectrics^6,9^. Furthermore, existing approaches for modeling material removal either rely on simplified damage criteria or focus on localized interaction volumes without providing a consistent link between optical excitation, energy transfer, and resulting ablation geometry.

In this work, we address this gap by developing a unified modeling framework that consistently couples the transient optical response, the electron dynamics, and the subsequent thermal evolution of the material. The optical properties are described using a hybrid Lorentz–Lorentz–Drude formalism, which enables the simultaneous consideration of bound valence electrons, localized excited states, and free carriers, thereby capturing the transition between different electronic regimes during excitation. The resulting energy deposition is directly coupled to a modified two-temperature model incorporating electron-density-dependent material parameters derived from first-principles calculations^9^ and represented in the present model by analytical fit functions. This approach allows for a physically consistent description of energy transfer from the electronic system to the lattice over a wide range of excitation conditions.

The predictive capability of the model is assessed through direct comparison between simulated and experimentally measured ablation geometries, allowing evaluation of morphological features and deviations arising from processes such as melt dynamics. This combined theoretical and experimental approach provides a coupled framework for analyzing ultrafast laser–matter interaction in fused silica and enables a deeper understanding of the mechanisms governing laser-induced material modification across different excitation regimes.

## Results

The investigated pulse durations of 200 fs, 1 ps, and 10 ps were selected with respect to the characteristic timescale of electron–phonon coupling, which governs the energy transfer from the excited electronic system to the lattice. In fused silica, this relaxation process occurs on a sub-picosecond timescale and depends on the excitation level and resulting carrier density. First-principles calculations report electron–phonon relaxation times of approximately 350 fs at moderate excitation and below 700 fs even at high excitation levels^9^, while characteristic coupling times on the order of 1 ps are commonly employed in modeling approaches and supported by previous studies^13,14^. Accordingly, three interaction regimes are considered: at 200 fs, the pulse duration remains well below the electron–phonon coupling time, resulting in a strongly non-equilibrium excitation; at 1 ps, it becomes comparable to the electron–phonon coupling time, marking a transition regime; and at 10 ps, it significantly exceeds the electron–phonon coupling time, leading to a thermally dominated response where electron and lattice subsystems approach quasi-equilibrium during energy deposition.

**Fig. 1:**
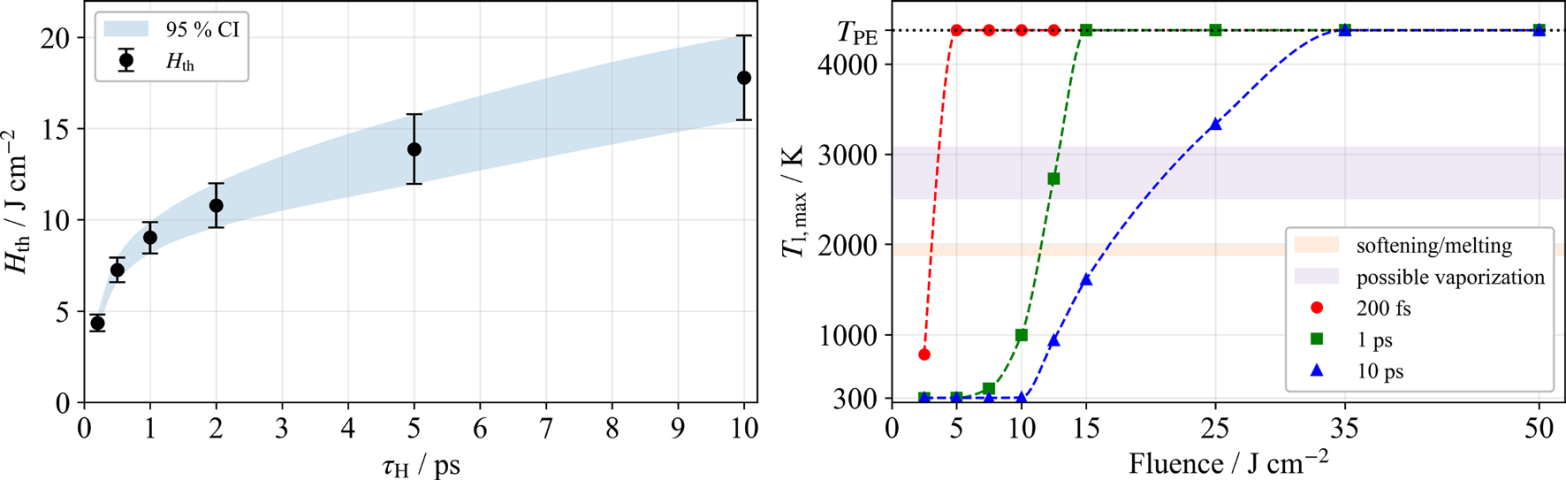
Experimental ablation thresholds and simulated maximum lattice temperatures. Left: Experimentally determined single-pulse ablation threshold $H_{\textrm{th}}$ as a function of pulse duration $\tau _{\textrm{H}}$. The shaded region indicates the confidence interval of the fit. Right: Simulated maximum lattice temperature $T_{\mathrm {l,\max }}$ in the central column as a function of fluence for pulse durations of 200 fs, 1 ps, and 10 ps. The horizontal dashed line indicates the phase-explosion surrogate criterion $T_{\textrm{PE}}$ used for material removal in the model. The shaded regions indicate the softening/melting range and a possible vaporization range.

The investigated fluence ranges were selected based on experimentally determined single-pulse ablation thresholds and the model-internal temperature criterion for material removal. The experimentally determined threshold increases with pulse duration, from approximately 4.5 J/cm^2^ at 200 fs to about 9 J/cm^2^ at 1 ps and 18 J/cm^2^ at 10 ps (Fig. 1, left). This trend reflects the reduced efficiency of ultrafast energy coupling with increasing pulse duration and provides the experimental basis for selecting the fluence range considered in the following sections.

The simulated maximum lattice temperatures indicate that the phase-explosion surrogate criterion $T_{\textrm{PE}}$, used as the material-removal criterion in the model, is reached close to the experimentally determined ablation threshold for 200 fs pulses (Fig. 1, right). For 1 ps and 10 ps pulses, however, experimentally measurable material removal occurs at fluences for which the simulated lattice temperature remains below $T_{\textrm{PE}}$. In these regimes, the maximum lattice temperatures reach ranges where possible vaporization, near-surface nucleation, or thermo-mechanical material removal may contribute to the experimentally observed ablation onset. Accordingly, the experimentally determined ablation threshold represents the first measurable permanent material removal, whereas $T_{\textrm{PE}}$ defines the model-internal criterion for phase-explosion-like ablation. The detailed comparison of ablation geometries in the following sections is therefore focused on fluence values for which the model predicts material removal according to this criterion, while identical fluences, particularly 35 and 50 J/cm^2^, are retained wherever possible to compare the material response across pulse durations.

### Femtosecond regime

**Fig. 2:**
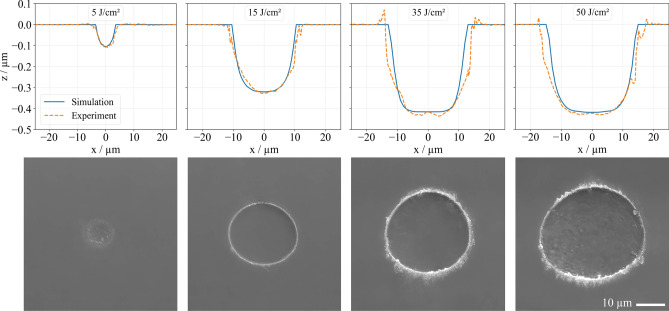
Fluence-dependent ablation profiles obtained for a pulse duration of 200 fs. **Top row:** Comparison between simulated (solid blue lines) and experimentally measured (dashed orange lines) ablation profiles showing the ablation depth *z* as a function of the lateral coordinate *x* for fluences of 5, 15, 35, and 50 J/cm^2^. **Bottom row:** Corresponding SEM images of the ablation geometries.

The ablation profiles obtained at a pulse duration of 200 fs exhibit a clear dependence on the applied fluence. Already at 5 J/cm^2^, a defined material removal is observed, exhibiting a profile that closely follows the Gaussian spatial intensity distribution of the incident beam (Fig. 2, top row). A very good agreement between simulation and experiment is maintained over the entire parameter range, while both the depth and the lateral extent of the ablation profile increase with increasing fluence.

**Fig. 3:**
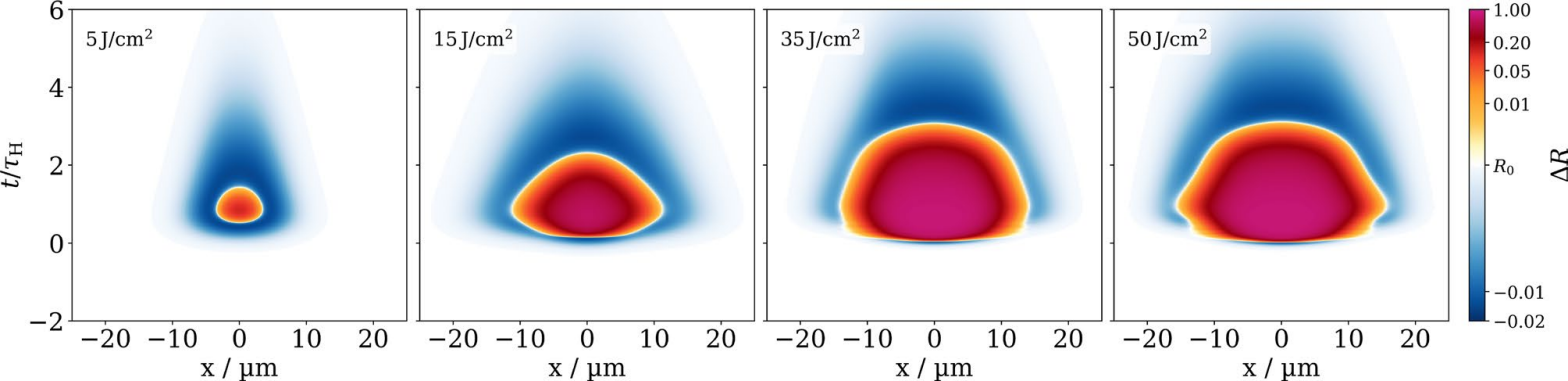
Transient change in reflectivity $\Delta R$ relative to the initial reflectivity $R_0$ = 0.03 as a function of lateral position *x* and normalized time $t/\tau _\textrm{H}$ for a pulse duration $\tau _\textrm{H}$ of 200 fs at different fluences. The temporal coordinate is referenced to the peak of the Gaussian pulse, which is located at $t\,=\,0$.

With increasing fluence, the initially Gaussian-like ablation profile gradually changes in shape. While the ablation profile at low fluence exhibits a pronounced central minimum, a progressive flattening of the profile bottom is observed at intermediate fluence, eventually leading to the formation of a flat-bottomed ablation geometry at higher fluences. Further increase in fluence does not result in a continued deepening of the ablation profile, but instead primarily leads to a lateral expansion of the ablation geometry (Fig. 2, top row). A quantitative comparison of ablation depth and diameter is presented below. This behavior can be directly linked to a fluence-dependent modification of the optical response. With increasing excitation, the transient reflectivity rises significantly, strongly limiting the fraction of energy coupled into the material. As a result, the incident energy is increasingly reflected rather than absorbed, leading to a saturation of the deposited energy in the beam center (Fig. 3). The transition from a Gaussian-like profile to a flat-bottomed ablation geometry is therefore not primarily caused by thermal diffusion or melt transport, but by a saturation of energy coupling induced by the strongly increased reflectivity.

Similar transitions from Gaussian-shaped profiles to flat-bottomed ablation geometries at higher fluences have been reported in previous experimental studies on dielectric materials^15,16^. While these works describe the phenomenological behavior, the simulations presented here provide a consistent physical interpretation by directly linking the observed saturation to the transient optical response of the material.

The corresponding SEM images reveal that the ablation geometries remain comparatively smooth over a wide fluence range, particularly at low and intermediate fluences (Fig. 2, bottom row). Although the deposited energy leads to transient melting and material removal via ablation processes, the morphology of the remaining material is only weakly affected by hydrodynamic motion. This indicates that, while strong hydrodynamic expansion occurs in the ablated material, the lateral redistribution of the residual melt layer is limited under these conditions. As a result, only minor deviations between simulated and experimental profiles are observed. At higher fluences, a slight increase in surface roughness and a pronounced material accumulation at the edges of the ablation geometry becomes visible. This feature is not captured by the simulation and can be attributed to lateral melt displacement driven by pressure gradients associated with the ablation process, which are not included in the present modeling approach.

### Picosecond regime

**Fig. 4:**
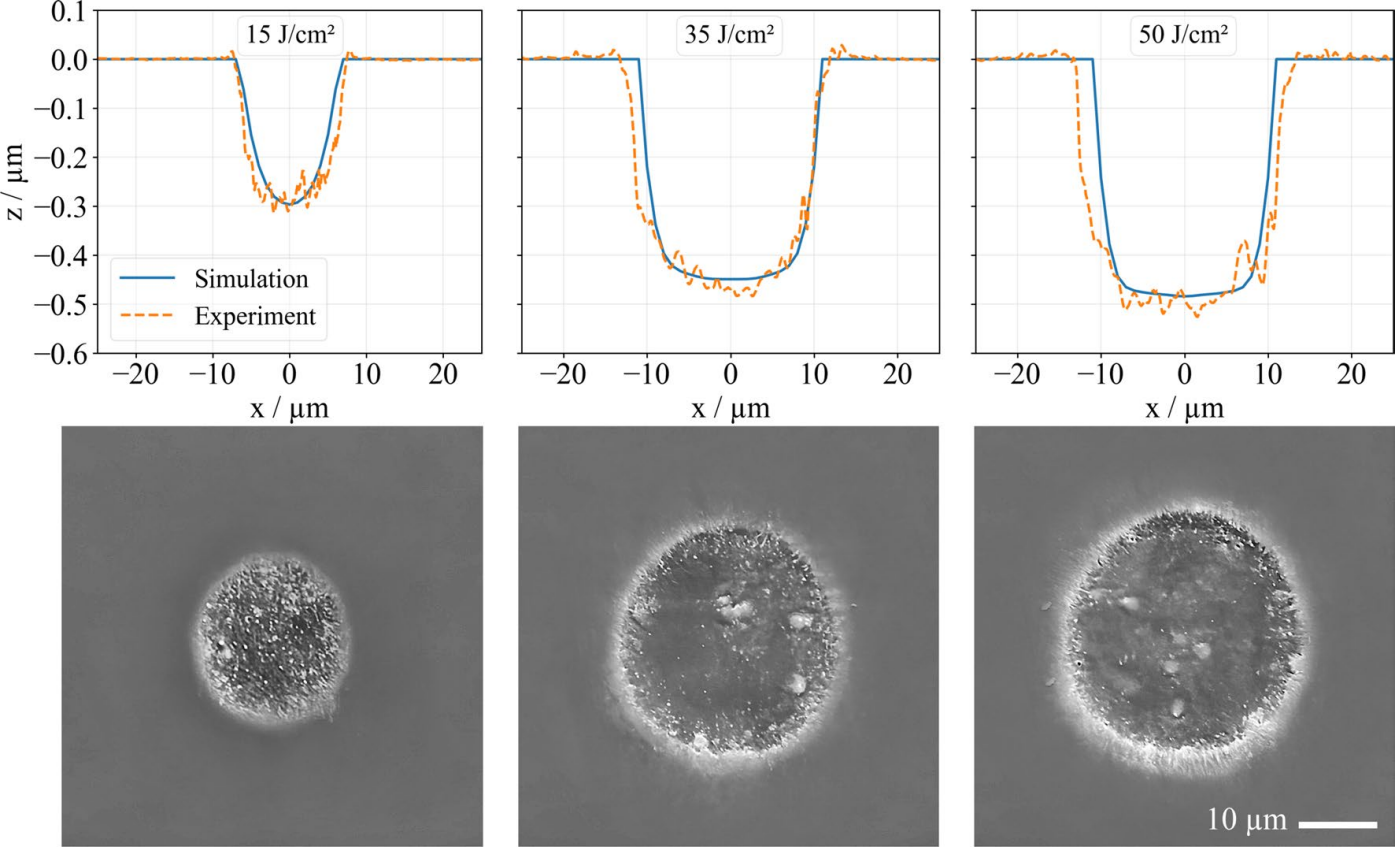
Fluence-dependent ablation profiles obtained for a pulse duration of 1 ps as a function of fluence. **Top row:** Comparison between simulated (solid blue lines) and experimentally measured (dashed orange lines) ablation profiles showing the ablation depth *z* as a function of the lateral coordinate *x* for fluences of 15, 35, and 50 J/cm^2^. **Bottom row:** Corresponding SEM images of the ablation geometries.

The ablation profiles obtained at a pulse duration of 1 ps exhibit the same general trends observed in the femtosecond regime. A defined material removal is obtained at 15 J/cm^2^, exhibiting a profile that still resembles the Gaussian spatial intensity distribution. With increasing fluence, both the depth and the lateral extent of the ablation geometry increase, while the agreement between simulated and experimental profiles remains generally good over the investigated parameter range (Fig. 4, top row). At higher fluences, a transition toward a flat-bottomed ablation geometry is observed, consistent with the behavior discussed for the femtosecond regime. While the ablation depth continues to increase with fluence, the increase becomes progressively less pronounced, and the ablation geometry expands predominantly in the lateral direction. This behavior indicates a progressively limited energy coupling in the beam center, consistent with the reflectivity-driven mechanism identified in the previous section.

In contrast to the femtosecond regime, the ablation geometries exhibit pronounced surface inhomogeneities across the entire modified region. Irregular, spike-like surface structures are visible in the SEM images and are reflected in pronounced fluctuations of the experimental profiles within the central region compared to the smoother profiles observed for 200 fs pulses (Figs. 2 and 4). These structures are consistent with resolidified micro-protrusions formed by melt deformation during material removal. In this interpretation, the interaction between ejected material and the underlying transient melt layer may promote localized melt displacement, elongation, and subsequent resolidification, resulting in a non-uniform surface morphology characterized by spike-like features. Similar hydrodynamic deformation and breakup processes have been predicted in simulations of ultrafast laser ablation under strong pressure gradients and rapid phase transitions^17^. In addition, experimental studies on femtosecond laser irradiation of metals have reported dense arrays of micro- and nano-protrusions that were attributed to melt-pool instabilities and rapid resolidification processes^18^. Although these studies were performed on metallic systems, they provide a useful phenomenological reference for interpreting the melt-related surface features observed here.

The deviations between simulation and experiment in the flank regions remain comparable to those observed in the femtosecond regime and can be attributed to lateral melt displacement driven by pressure gradients during the ablation process. In contrast, the additional inhomogeneities observed within the ablation geometry indicate that hydrodynamic effects increasingly influence the local surface morphology. These effects are not captured by the present modeling approach, which does not account for melt dynamics, but remain limited in magnitude and do not significantly affect the overall agreement in terms of ablation profile shape. The appearance of such melt-related surface features suggests a gradual transition toward a regime in which hydrodynamic processes increasingly contribute to the final morphology, which is further examined for longer pulse durations in the following section.

### Thermally dominated regime

**Fig. 5:**
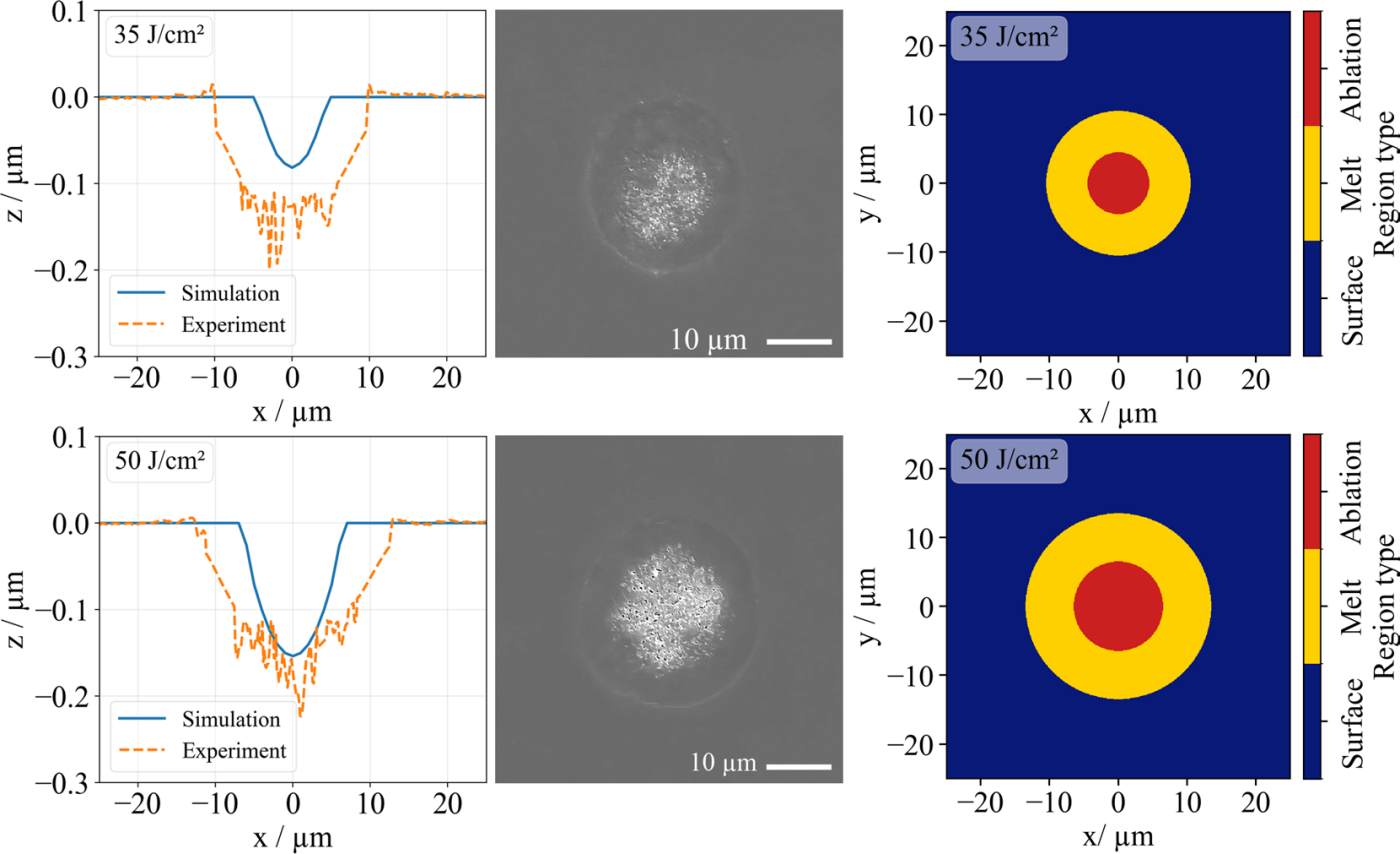
Ablation profiles, surface morphologies, and simulated material regions for a pulse duration of 10 ps at fluences of 35 and 50 J/cm^2^. **Left column:** Comparison between simulated (solid blue lines) and experimentally measured (dashed orange lines) ablation profiles. **Middle column:** Corresponding SEM images of the ablation geometries. **Right column:** Simulated material regions indicating the spatial extent of surface (blue), melt (yellow), and ablation (red), based on the applied temperature criteria.

The ablation profiles obtained at a pulse duration of 10 ps differ fundamentally from those observed in the femtosecond and picosecond regimes. Evaluating the findings exclusively on the basis of the cross-sectional profiles, substantial differences between simulation and experiment are evident (Fig. 5, left column). At 35 J/cm^2^, the simulated profile underestimates both the depth and the lateral extent of the experimentally observed structure, whereas at 50 J/cm^2^ the depth is reproduced more closely while the lateral extent remains significantly underestimated. This behavior indicates that the final surface morphology cannot be described by the ablation criterion alone. A more detailed analysis reveals that the experimental profiles consist of two distinct regions. The outer region is characterized by comparatively smooth and gradually varying flanks, whereas the central region exhibits pronounced fluctuations and a strongly irregular surface morphology. This distinction is consistently observed for both investigated fluences and is directly reflected in the corresponding SEM images (Fig. 5, middle column), which reveal a clear transition from a smooth outer morphology to a highly structured inner region. A comparison with the simulation results shows that the spatial extent of the rough inner region corresponds closely to the area identified as ablation by the model. This agreement indicates that the simulation captures the region in which the ablation criterion is fulfilled. In contrast, the smooth outer region observed experimentally is not represented in the simulated ablation profiles and therefore cannot be attributed to direct material removal by ablation.

The origin of this additional region becomes apparent when considering the simulated material states. The model predicts a pronounced melt zone surrounding the ablation region, extending significantly beyond the area where the ablation criterion is fulfilled (Fig. 5, right column). This indicates that a substantial volume of material is heated above the melting temperature without undergoing ablation. The threshold analysis in Fig. 1 supports this interpretation, showing that, for 10 ps pulses, experimentally measurable material removal can occur at fluences below the model-internal phase-explosion criterion $T_{\textrm{PE}}$. The spatial correlation between this melt region and the smooth outer morphology observed experimentally suggests that this part of the structure is associated with thermally modified material outside the directly predicted ablation zone. The formation of the outer region is therefore attributed to processes that are not explicitly resolved by the present model, including sub-$T_{\textrm{PE}}$ thermal material removal, possible vaporization or near-surface nucleation, and subsequent melt smoothing and resolidification. This interpretation differs fundamentally from the smooth ablation geometries observed in the femtosecond regime. For 200 fs pulses, the comparatively homogeneous morphology corresponds closely to the directly predicted ablation region and is primarily determined by localized energy deposition.

In contrast, the smooth outer region observed at 10 ps extends significantly beyond the simulated ablation zone and spatially correlates with the predicted melt region, indicating that its formation is associated with thermally mediated melt modification rather than direct phase-explosion-like ablation. A simple outward expulsion of melt driven by ablation-induced pressure gradients appears unlikely, as no significant accumulation of resolidified droplets or pronounced rim formation is observed outside the modified region. In addition, the boundary of the structure remains comparatively smooth, in contrast to the more pronounced rim features observed at shorter pulse durations (Fig. 5, middle column). These observations suggest that the transient melt layer remains coupled to the material-removal region, but the underlying dynamics cannot be resolved within the present independent-column model. Possible contributions include deformation of the molten layer, localized melt displacement along the evolving material-removal front, and subsequent resolidification. While this interpretation remains qualitative, it is consistent with both the observed morphology and the spatial relation between the ablation and melt regions predicted by the model.

In addition to these lateral redistribution effects, the pronounced roughness within the central region indicates the increasing influence of hydrodynamic instabilities within the melt layer itself. The strong fluctuations observed in the experimental profiles suggest that the melt undergoes dynamic deformation during and after material removal. Such processes are not captured by the present modeling approach, which neglects melt dynamics and therefore cannot reproduce the observed surface morphology. Consequently, two limitations of the model become apparent in this regime. First, the absence of hydrodynamic processes within the transient melt layer prevents the reproduction of the irregular surface structure in the central region. Second, the lack of lateral coupling between neighboring regions inhibits the description of melt redistribution beyond the ablation zone. Overall, the results demonstrate that, at a pulse duration of 10 ps, the final surface morphology results from the combined action of ablation, melting, and hydrodynamic material redistribution, which extends beyond the region defined by the ablation criterion.

### Quantitative comparison of ablation depth and diameter

**Fig. 6:**
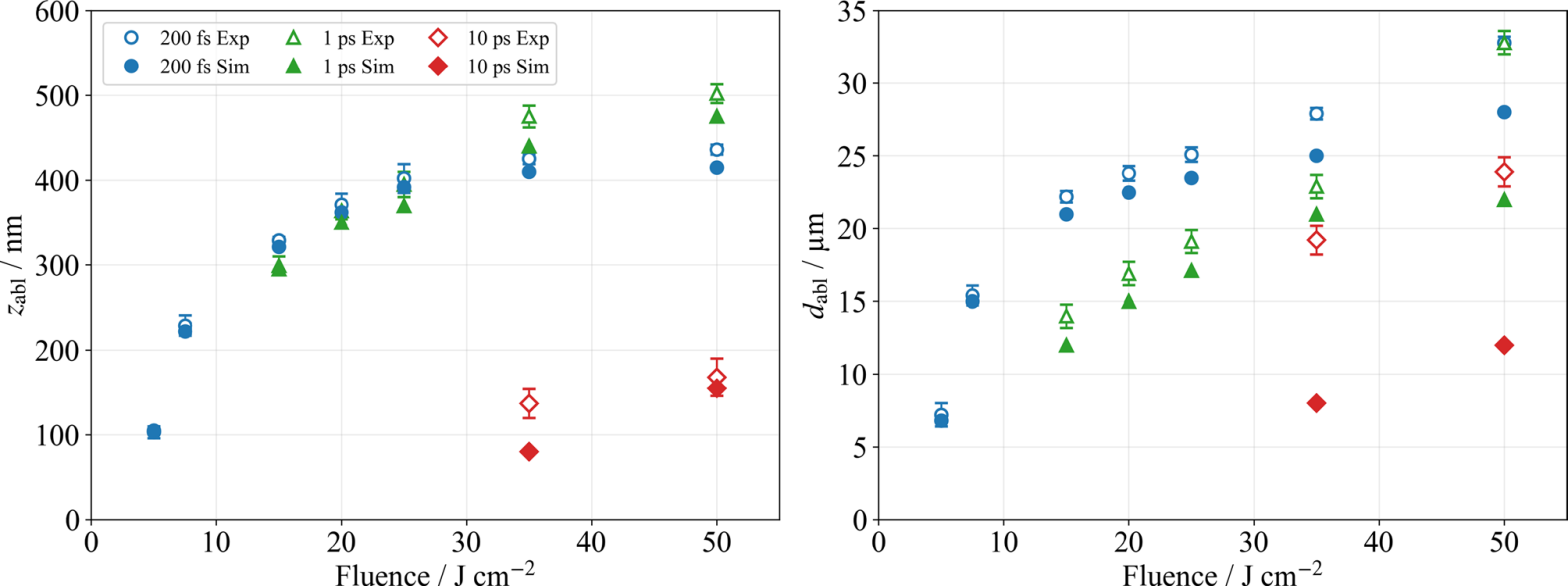
Simulated and experimentally measured ablation depth (left) and ablation diameter (right) as a function of fluence for pulse durations of 200 fs, 1 ps, and 10 ps.

A quantitative comparison between simulated and experimentally determined values of $z_{\text {abl}}$ and $d_{\text {abl}}$ is illustrated in Fig. 6 for the investigated pulse durations and fluences for which the model predicts material removal according to the applied $T_{\textrm{PE}}$ criterion. The experimental values are shown together with their standard deviations, enabling direct assessment of the agreement between simulation and experiment. The threshold analysis further shows that the experimentally determined ablation threshold and the model-internal material-removal criterion are not identical quantities. The experimental threshold corresponds to the onset of permanent measurable material removal, as determined from the lateral extent of ablation structures. In contrast, the temperature criterion $T_{\textrm{PE}}$ used in the model represents a phase-explosion surrogate for direct material removal. Consequently, material modification or removal at fluences below $T_{\textrm{PE}}$ may occur through processes not explicitly included in the present framework, such as possible vaporization, near-surface nucleation, or thermo-mechanical material removal. This distinction is particularly relevant for the longer pulse durations, where deviations between the experimental threshold and the model-internal criterion become more pronounced.

For a pulse duration of 200 fs, the simulated values of $z_{\text {abl}}$ closely reproduce the experimental results over the entire investigated fluence range, while $d_{\text {abl}}$ remains within a limited deviation range. At 50 J/cm^2^, for example, the experimentally determined ablation depth is reproduced within approximately 5 %. Overall, both the depth and the lateral extent of the ablation geometry are described with good agreement in the femtosecond regime.

At a pulse duration of 1 ps, the simulated values of $z_{\text {abl}}$ remain consistent with the experimental results within a comparable deviation range. In contrast, $d_{\text {abl}}$ becomes systematically underestimated by the model, particularly at higher fluences. This deviation indicates that lateral material modification increasingly extends beyond the region described by the temperature-based phase-explosion criterion. At identical fluence, the experimentally observed ablation depths exceed those obtained for 200 fs pulses, indicating an increased effective energy coupling into the material at picosecond pulse durations.

This behavior is consistent with the reduced influence of transient reflectivity effects discussed in the previous section.

For a pulse duration of 10 ps, substantially larger deviations between simulation and experiment are observed. While the simulated values of $z_{\text {abl}}$ remain in qualitative agreement with the experimental results, $d_{\text {abl}}$ is strongly underestimated over the investigated fluence range. These deviations are consistent with the observations discussed for the thermally dominated regime, where the experimentally observed outer regions originate from melt-mediated material redistribution beyond the directly simulated ablation zone.

Overall, the quantitative comparison demonstrates that the model reproduces the main ablation trends in the femtosecond regime and remains predictive for $z_{\text {abl}}$ in the picosecond regime. With increasing pulse duration, melting and hydrodynamic material transport increasingly influence the final morphology, leading to larger deviations, particularly in the lateral extent of the ablation geometry. These effects are not captured by the present modeling approach and therefore define its current limits of applicability.

## Discussion

The objective of this study was to develop and evaluate a physically consistent model for the description of ultrashort laser–matter interaction in fused silica, with particular focus on the transition from non-equilibrium to thermally dominated regimes. To this end, a coupled opto-electronic and two-temperature modeling approach was applied and systematically compared with experimental ablation geometries over a wide range of pulse durations and fluences.

The results show that the model reproduces the main experimental trends in the femtosecond regime. Both the ablation depth and diameter are reproduced within a limited deviation range, supporting the applicability of the coupled description under strongly non-equilibrium conditions without uniquely validating each internal model parameter. In this regime, the interaction is primarily dominated by non-equilibrium dynamics, and the influence of melt-mediated processes on the final surface morphology remains limited.

With increasing pulse duration, a gradual transition in the dominant interaction mechanisms is observed. In the picosecond regime, the model continues to describe the ablation depth within a comparable deviation range, while systematic deviations in the ablation diameter become apparent. At identical fluence, an increased ablation depth is observed compared to the femtosecond regime, indicating a more efficient energy coupling into the material. This behavior is consistent with the reduced influence of transient reflectivity effects and the increasing contribution of energy transfer to the lattice during the pulse duration.

At a pulse duration of 10 ps, the limitations of the present modeling approach become evident. While the simulated ablation region corresponds to the central part of the experimentally observed structures, the overall geometry cannot be described solely by the ablation criterion. The experimental results reveal an additional outer region that originates from melt-mediated material redistribution. This contribution is not captured by the current model and leads to significant deviations, particularly in the lateral extent of the ablation geometry.

These observations highlight two principal limitations of the present approach. First, the model does not account for hydrodynamic processes within the transient melt layer, which are responsible for the formation of surface instabilities and the resulting roughness in the ablated region. Second, lateral heat diffusion and hydrodynamic coupling between neighboring regions are not considered in the independent-column approximation, preventing the description of thermally mediated melt redistribution beyond the directly simulated ablation zone. The inclusion of hydrodynamic effects represents a possible extension of the model. In principle, the incorporation of melt dynamics on a per-column basis could improve the description of surface morphology. However, such an approach would significantly increase the computational effort and must therefore be evaluated with respect to its benefit in relation to the intended application. A full description of lateral hydrodynamic interactions would require multidimensional extensions beyond the scope of the present framework.

Additional limitations arise from the effective treatment of the opto-electronic excitation dynamics. The bandgap is kept constant, while the Drude collision time, effective electron mass, avalanche ionization coefficient, trapping time, recombination coefficient, and the parameters of the localized-state Lorentz contribution are represented by literature-based or phenomenological values. Reported values for several of these quantities cover broad ranges and may depend on wavelength, pulse duration, intensity, carrier density, electron temperature, the experimental observable, and the specific model used for their extraction. The selected values were kept fixed across all investigated pulse durations and fluences and were not adjusted individually to reproduce the respective ablation geometries. Nevertheless, agreement with the final ablation profiles evaluates the integrated response of the coupled framework but does not uniquely identify each microscopic parameter. Partially compensating influences between effective parameters therefore cannot be excluded on the basis of the present ex-situ measurements.

The comparatively short time available for carrier multiplication at 200 fs suggests that the influence of uncertainties in the avalanche contribution may be lower than at longer pulse durations, although this cannot be quantified from the present data. In addition, the transient optical attenuation and depth-dependent energy deposition are included through the dynamically evaluated dielectric response, whereas full nonlinear beam propagation, including self-focusing, plasma-induced defocusing, and transverse beam reshaping, is not resolved. A purely numerical parameter variation would quantify the mathematical response of the model to selected parameter ranges but would not establish whether the resulting transient optical and electronic dynamics are physically correct. Their individual assessment requires time- and spatially resolved constraints on the transient refractive index, extinction coefficient, and reflectivity, for example by pump–probe ellipsometry and reflectometry below and close to the ablation threshold. Within these limitations, the model provides an effective, spatially and temporally resolved description of the coupled optical and thermodynamic response of the material.

The specific contribution of the present framework lies in coupling the hybrid dielectric response, including localized electronic states, to an electron-density-dependent two-temperature description, rather than in claiming unique determination of all underlying opto-electronic parameters. This capability provides a foundation for further investigations into fundamental laser–matter interaction processes. In particular, the model can be extended to study the influence of pulse shaping, spatial beam modulation, and wavelength variation on the energy deposition and resulting material modification. In regimes where hydrodynamic effects remain limited, the approach enables predictive simulations of ablation geometries, which is of direct relevance for micro- and nano-structuring applications.

Furthermore, the framework offers the potential to investigate multi-pulse interactions, including incubation and heat accumulation effects. By extending the model to sequences of pulses with defined repetition rates, it becomes possible to analyze the evolution of material properties and surface geometry under repeated irradiation. Such extensions would provide a direct link between fundamental interaction mechanisms and application-oriented processing conditions.

In summary, the presented model provides an effective description of ultrashort laser ablation in dielectric materials within the non-equilibrium and transition regimes, while its applicability becomes limited in thermally dominated regimes due to the increasing importance of melt dynamics. The results establish both the capabilities and the boundaries of the approach and provide a basis for future developments aimed at extending the model toward more complex interaction scenarios.

## Methods

### Theoretical model

The theoretical framework employed in this study combines a transient optical–electronic excitation model with a density-dependent two-temperature description of the subsequent thermal response. A detailed formulation of the model is provided in the Supplementary Information.

The transient optical response of fused silica is described using a hybrid dielectric function that accounts for contributions from bound valence electrons, electrons in localized states, and free conduction-band electrons. This approach enables a consistent representation of the evolving electronic structure during ultrafast excitation, capturing the transition from bound to free-electron-dominated behavior. The corresponding electron dynamics are governed by rate equations including primary photoexcitation, avalanche ionization, as well as trapping and recombination processes, which determine the time- and depth-dependent conduction-band electron density.

The resulting energy deposition is obtained from the transient optical properties and directly coupled to a modified two-temperature model. In contrast to classical formulations, the electron subsystem is described using electron-density-dependent material parameters. In particular, the electron heat capacity and the electron–phonon coupling factor are expressed as functions of the transient conduction-band electron density based on first-principles calculations reported by Tsaturyan et al.^9^ and represented in the present model by analytical fit functions. This enables a physically consistent description of the energy transfer between the electronic and lattice subsystems under strongly non-equilibrium conditions.

The thermal response is calculated using a one-dimensional formulation along the depth coordinate, while the lateral energy distribution is accounted for using an independent column approximation, allowing reconstruction of spatially resolved ablation geometries. In addition to the predicted ablation geometry, the applied temperature criteria enable identification of thermally modified regions associated with melting and direct material removal.

### Experimental details

An amplified solid-state laser system (Carbide CB3-80W, Light Conversion) was employed, operating at a wavelength of 1030 nm and providing linearly polarized pulses with a tunable pulse duration between 200 fs and 10 ps. The laser beam exhibited a near-Gaussian spatial intensity profile ($M^2\,\le \,$1.13) and was focused onto the sample surface using an f-theta lens, resulting in a beam waist radius of 14 μm.          

The investigated material was amorphous fused silica (SiO_2_), characterized by a wide bandgap of approximately 9 eV^19^. The pulse duration was varied from 200 fs to 10 ps, and the applied single-pulse fluence ranged up to 50 J/cm^2^, consistent with the parameter range considered in the simulations. The single-pulse ablation thresholds were determined using the diameter-squared method introduced by Liu^20^, in which the squared diameter of the modified region is evaluated as a function of pulse energy or peak fluence and extrapolated to $D^2$ = 0.

**Fig. 7:**
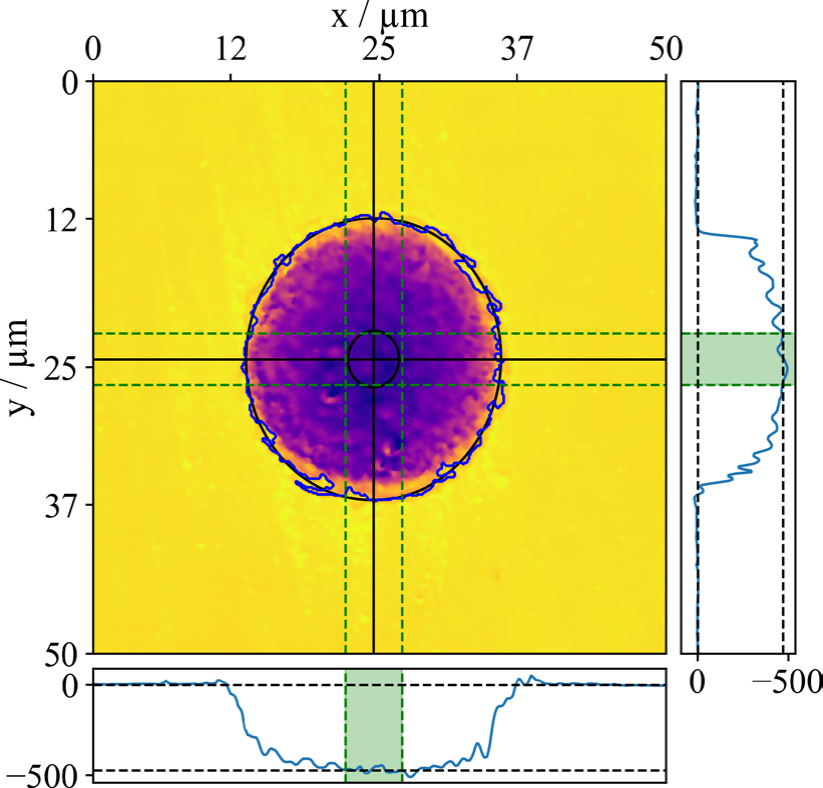
Exemplary evaluation of ablation geometry from confocal laser scanning microscopy for a pulse duration of 1 ps at a fluence of 35 J/cm^2^.

The resulting surface morphologies were analyzed using confocal laser scanning microscopy (Olympus Lext 3D OLS4100) to obtain quantitative topographical information. Cross-sectional profiles were extracted in orthogonal directions and averaged to determine representative ablation geometries. To illustrate the evaluation procedure, an exemplary topographical measurement obtained by confocal laser scanning microscopy is shown in Fig. 7. The ablation diameter $d_{\text {abl}}$ is determined from the lateral extent of the modified region, while the ablation depth $z_{\text {abl}}$ is extracted from averaged cross-sectional profiles along orthogonal directions. To reduce the influence of local surface fluctuations, the depth is evaluated within a defined central region. These experimental profiles were used for direct comparison with the simulated ablation depths and profiles. In addition, scanning electron microscopy (SEM, Jeol JSM-6512 V) was used for qualitative assessment of the surface morphology, particularly to identify melt-related features and hydrodynamic signatures not explicitly captured by the model.

## Supplementary Information

Supplementary Information.

## Data Availability

The datasets used and/or analysed during the current study are available from the corresponding author on reasonable request.
