# Supplementary material for: Modeling ultrafast laser excitation of fused silica with a hybrid Lorentz–Drude dielectric response and density-dependent two-temperature model
Source: Sci Rep. 2026 Jul 27;16:23358. doi: 10.1038/s41598-026-63133-7 (PMC13408711; doi:10.1038/s41598-026-63133-7)
Supplement: Supplementary file 1 — Supplementary Information. [file 41598_2026_63133_MOESM1_ESM.pdf]

Supplementary Information

Modeling ultrafast laser excitation of fused silica with a hybrid Lorentz–Drude dielectric response and density-dependent two-temperature model

Daniel Metzner[1,*], Philipp Rebentrost[1], Manuel Pfeiffer[1], Peter Lickschat[1], Jonas Opitz[1], and Steffen Weißmantel[1]

[1]University of Applied Sciences Mittweida, Laserinstitut Hochschule Mittweida, Mittweida, 09648, Germany
*metzner@hs-mittweida.de

S1 Laser field model

The incident laser pulse is modeled as a linearly polarized ultrashort pulse with a Gaussian temporal envelope. Since the excitation dynamics of the optical–electronic model are driven by the electric field amplitude, the laser pulse is introduced through its electric field rather than through the intensity.

The electric field of the laser pulse $\vec{E}(t)$ can be written as an oscillating carrier field with a Gaussian envelope

$$\vec{E}(t) = E_0 \exp\left(-2\ln(2)\frac{t^2}{\tau_{\mathrm{H}}^2}\right)\cos(\omega t), \tag{1}$$

where E_0 denotes the peak electric field amplitude, t is the time, ω is the optical angular frequency of the laser, and τ_{H} represents the temporal full width at half maximum of the pulse envelope. The connection between the electric field and the optical intensity follows from the time-averaged Poynting vector

$$I(t) = \frac{1}{2} n\,\varepsilon_0\, c\,\vec{E}_{\mathrm{env}}^2(t), \tag{2}$$

where c denotes the speed of light in vacuum, ε_0 is the vacuum permittivity, n is the refractive index of the material, and $\vec{E}_{\mathrm{env}}(t)$ represents the varying envelope of the electric field. Substituting the Gaussian envelope into Eq. (2) yields the temporal intensity distribution of the laser pulse

$$I(t) = I_0 \exp\left(-4\ln(2)\frac{t^2}{\tau_{\mathrm{H}}^2}\right), \tag{3}$$

where I_0 denotes the peak intensity of the laser pulse. The corresponding peak electric field amplitude is related to the peak intensity through

$$E_0 = \sqrt{\frac{2 I_0}{n\,\varepsilon_0\, c}}, \tag{4}$$

which provides the electric field amplitude used as the driving quantity in the excitation model.

The spatial intensity distribution at the surface is assumed to follow a Gaussian beam profile

$$I(r) = I_0 \exp\left(-2\frac{r^2}{w_0^2}\right), \tag{5}$$

where r denotes the lateral radial coordinate with respect to the beam center, w_0 the beam waist radius defined as the $1/e^2$ radius of the lateral intensity distribution. Accordingly, approximately 86 % of the total pulse energy is contained within the radius w_0. Combining the spatial and temporal dependencies yields the complete spatially and temporally intensity distribution of the incident laser pulse

$$I(r,t) = I_0 \exp\left(-2\frac{r^2}{w_0^2}\right)\exp\left(-4\ln(2)\frac{t^2}{\tau_{\mathrm{H}}^2}\right). \tag{6}$$

The experimentally controlled parameter in laser processing is typically the laser fluence, defined as the time integral of the intensity

$$H(r) = \int_{-\infty}^{\infty} I(r,t)\, dt = H_0 \exp\left(-2\frac{r^2}{w_0^2}\right), \tag{7}$$

where H_0 denotes the peak fluence at the beam center. Here, ($r \geq 0$) denotes the radial distance from the beam center in the rotationally symmetric Gaussian beam profile. In the column-based reconstruction and in all cross-sectional profiles, the lateral coordinate is denoted by x, allowing positive and negative positions relative to the beam center.

This laser field description provides the input for the optical-electronic excitation model described in the following sections, where the electric field amplitude governs the photoexcitation and electron dynamics in the material.

S2 Electron excitation dynamics

The laser-induced excitation dynamics are described by the temporal evolution of the conduction band electron density n_e. In the present model, electrons are transferred from the valence band into the conduction band by primary photoexcitation and secondary avalanche ionization, while trapping and electron–hole recombination act as loss mechanisms. To account for the finite number of excitable electrons in the material, both excitation channels are limited by a saturation term.

The temporal evolution of the conduction band electron density is therefore written as [1–4]

$$\frac{dn_e}{dt} = W_{PI}\left(I, \gamma, \widetilde{E}_{gap}\right)\left(1 - \frac{n_e}{n_{max}}\right) + W_{AI}(n_e, I)\left(1 - \frac{n_e}{n_{max}}\right) - R_{rec} - R_{trap}, \tag{8}$$

where W_{PI} denotes the primary photoexcitation rate, W_{AI} the avalanche ionization rate, R_{rec} the recombination loss term, and R_{trap} the trapping loss term. The saturation term $(1 - n_e/n_{max})$ ensures that the conduction band electron density does not exceed the maximum number of electrons that can be promoted from the valence band. For fused silica, the maximum excitable electron density is determined from the atomic density of SiO_2 and the number of excitable electrons per structural unit. Using an atomic density of $N_a = 22.04 \times 10^{27}\,m^{-3}$ and two excitable electrons, the maximum conduction band electron density becomes

$$n_{max} = 2N_a = 4.41 \times 10^{28}\,m^{-3}. \tag{9}$$

The primary photoexcitation rate W_{PI} is described within the Keldysh formalism [1], which continuously links the multiphoton ionization and tunneling ionization regimes. The corresponding Keldysh parameter is given by

$$\gamma = \frac{\omega\sqrt{m_{red} E_{gap}}}{e E_0}, \tag{10}$$

where ω is the optical angular frequency, E_{gap} is the band gap energy, e is the elementary charge, and E_0 is the electric field amplitude of the laser pulse. In the present model, E_{gap} is treated as a constant bandgap energy in the photoionization and avalanche-ionization terms. This represents an effective approximation of the excitation dynamics. Recent first-principles studies have shown that strong electronic excitation can lead to bandgap renormalization in fused silica and SiO_2, including bandgap narrowing, cohesion loss, and non-monotonic bandgap behavior at high electron temperatures[5–7]. Such effects can modify the nonlinear photoexcitation rate and may shift the onset of material modification, particularly close to the ablation threshold. However, the available data do not provide a simple monotonic correction that is valid over the full excitation range considered here. In particular, the bandgap evolution depends on the transient electron temperature, excitation density, structural relaxation, and the onset of thermodynamic instability. Therefore, bandgap renormalization is not included self-consistently in the present rate-equation model and is treated as a limitation of the current framework.

The reduced effective mass is approximated as $m_{red} \approx 0.5\, m_e$, following common practice in the literature[8]. In the limit of $\gamma \gg 1$, photoexcitation is dominated by multiphoton absorption, whereas $\gamma \ll 1$ corresponds to the tunneling regime. This transition between multiphoton ionization and tunneling ionization is illustrated in Fig. 1, which shows the excitation rates as a function of both laser intensity and the Keldysh parameter for a wavelength of 1030 nm and a bandgap of 9 eV. At low intensities (high γ), the multiphoton ionization rate dominates, while at high intensities (low γ),

tunneling ionization becomes the prevailing excitation mechanism. The full Keldysh formulation provides a continuous description across both regimes.

The secondary excitation process is described by avalanche ionization according to [2–4]

$$W_{AI}(n_e, I) = \alpha_i\, I(t)\, n_e, \tag{11}$$

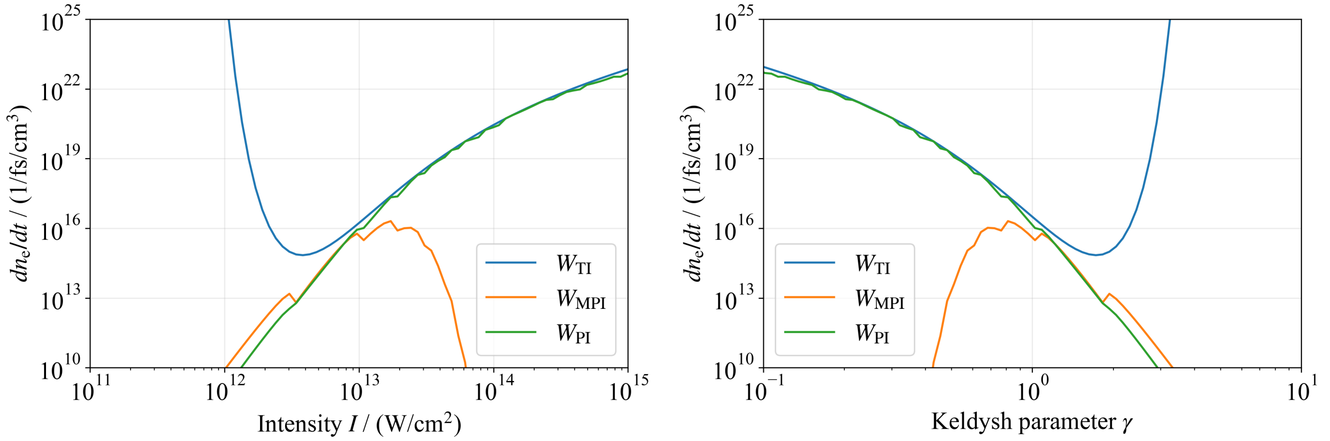

Figure 1. Excitation rates of primary photoionization in fused silica calculated using the Keldysh formalism. **Left**: Excitation rate as a function of laser intensity. **Right**: Excitation rate as a function of the Keldysh parameter γ. The asymptotic limits of multiphoton ionization (W_{MPI}) and tunneling ionization (W_{TI}) are compared with the full Keldysh rate (W_{PI}), illustrating the continuous transition between both regimes.

where α_i denotes the avalanche ionization coefficient and $I(t)$ is the local laser intensity. In the present simulations, a value of $\alpha_i = 4.0\,\mathrm{cm}^2/\mathrm{J}$ is used following established rate-equation modeling of fused silica[9]. The empirical basis of this coefficient can be traced to optical-breakdown measurements performed at a wavelength of 780 nm and interpreted within a single-rate model[10]. Its quantitative transfer to the present excitation conditions at 1030 nm is therefore not unique, because the effective avalanche contribution may depend on wavelength, pulse duration, intensity, collision dynamics, and the treatment of primary photoexcitation. Accordingly, α_i is treated here as an effective literature-based coefficient rather than as a universally valid microscopic rate. The same value is retained for all investigated pulse durations and fluences and was not adjusted to reproduce individual experimental ablation geometries. Because the time available for carrier multiplication is substantially shorter for 200 fs pulses than for the picosecond cases, the influence of uncertainties in the avalanche contribution may be lower in the shortest-pulse regime. However, this dependence cannot be quantified independently from the present ex-situ measurements.

The loss of conduction band electrons by trapping is described by a first-order process [11]

$$R_{trap} = \frac{n_e}{\tau_{trap}}, \tag{12}$$

where τ_{trap} denotes the characteristic trapping time. In the present model, $\tau_{trap} = 150\,\mathrm{fs}$ is used as an effective literature-based timescale[4, 11]. This term represents the transfer of quasi-free conduction-band electrons into localized states within the reduced rate-equation description. The effective trapping time may depend on excitation conditions, defect populations, and the transient material state and should therefore not be interpreted as a universal constant. Its value was kept fixed throughout all simulations and was not fitted separately for individual pulse durations or fluences.

Electron–hole recombination is described by a second-order loss term [2–4]

$$R_{rec} = \eta_{rec}\, n_e^2, \tag{13}$$

where η_{rec} is an effective bimolecular recombination coefficient. In the present model, a value of $\eta_{rec} = 1 \times 10^{-10}\,\mathrm{cm}^3\,\mathrm{s}^{-1}$ is used. This quantity is not treated as a universal material constant of fused silica, since reported recombination descriptions depend strongly on the excitation, transport, and trapping regime. It is therefore introduced as a phenomenological loss parameter and was kept unchanged across the complete simulation set. The present ex-situ ablation measurements do not permit the trapping and recombination contributions to be determined independently.

S3 Hybrid dielectric response model

The transient optical response of fused silica under ultrashort laser irradiation is described using a hybrid dielectric function that combines contributions from bound valence-band electrons, electrons trapped in localized states, and free conduction-band electrons. The total dielectric function is therefore written as

$$\varepsilon(\omega,t) = \varepsilon_\infty(t) + \varepsilon_{VB}(\omega,t) + \varepsilon_{loc}(\omega,t) + \varepsilon_e(\omega,t), \tag{14}$$

where ε_∞ denotes the high-frequency dielectric background, $\varepsilon_{\mathrm{VB}}$ represents the Lorentz response of bound valence-band electrons, $\varepsilon_{\mathrm{loc}}$ describes electrons occupying localized states, and ε_{e} corresponds to the Drude response of free conduction-band electrons. The relative contribution of these three terms evolves dynamically during laser excitation because the electron densities in the valence band, localized states, and conduction band change according to the rate-equation model described in Section S2. The relative weights of these dielectric contributions are not fixed model constants. Before excitation, the dielectric response is dominated by the valence-band Lorentz contribution and the equilibrium dielectric background. During laser excitation, the valence-band contribution decreases as electrons are removed from the valence band, while the localized-state Lorentz contribution and the Drude contribution increase according to the transient populations of localized and quasi-free electrons, respectively. Their relative importance therefore depends on time, depth, pulse duration, and fluence.

S3.1 Valence-band Lorentz oscillator

The optical response of bound valence-band electrons is described by a Lorentz oscillator [12,13]

$$\varepsilon_{\mathrm{VB}}(\omega,t) = s_{\mathrm{VB}}(t)\frac{\omega_{\mathrm{P,VB}}^2}{\omega_{0,\mathrm{VB}}^2-\omega^2-i\Gamma_{\mathrm{VB}}\omega}. \tag{15}$$

Here $s_{\mathrm{VB}}(t)$ denotes a time-dependent scaling factor representing the fraction of electrons remaining in the valence band, $\omega_{0,\mathrm{VB}}$ is the resonance frequency of the valence-band transition, Γ_{VB} is the damping constant, and $\omega_{\mathrm{P,VB}}$ is the oscillator strength parameter. The oscillator parameters themselves remain constant and represent the intrinsic optical response of the unexcited dielectric material. However, the effective contribution of this oscillator decreases during laser excitation because electrons are removed from the valence band and promoted into excited states. This effect is taken into account by scaling the oscillator amplitude according to the fraction of electrons that remain bound in the valence band

$$s_{\mathrm{VB}}(t) = \frac{n_{\mathrm{max}}-n_{\mathrm{tot}}(t)}{n_{\mathrm{max}}}, \tag{16}$$

where $n_{\mathrm{tot}} = n_{\mathrm{e}}+n_{\mathrm{loc}}$ denotes the total density of excited electrons. The parameters of the valence-band oscillator are chosen according to previously reported Lorentz models for fused silica.

Table 1. Parameters used for the valence-band Lorentz oscillator[8].

Parameter	Symbol	Value	Description
High-frequency offset	ε_∞	1.49	equilibrium dielectric background
Valence-band electron density	$n_a N_0$	$44\times10^{27}\,\mathrm{m}^{-3}$	density of participating valence electrons
Resonance energy	$\hbar\omega_{0,\mathrm{VB}}$	10.0 eV	valence-band transition energy
Damping constant	$\hbar\Gamma_{\mathrm{VB}}$	0.9 eV	spectral broadening

S3.2 Lorentz oscillator for localized electronic states

Electrons that become trapped in localized states during laser excitation remain optically active but behave as bound charges rather than free carriers. Their contribution to the dielectric response is therefore described by an additional Lorentz oscillator

$$\varepsilon_{\mathrm{loc}}(\omega,t) = s_{\mathrm{loc}}(t)\frac{\omega_{\mathrm{P,loc}}^2}{\omega_{0,\mathrm{loc}}^2-\omega^2-i\Gamma_{\mathrm{loc}}\omega}, \tag{17}$$

where the parameters are defined analogously to those introduced for the valence-band oscillator in Eq. (15), but here describing the optical response of electrons occupying localized states. In contrast to the valence-band oscillator, the contribution of this term appears only when localized electrons are present. This is implemented by scaling the oscillator amplitude according to the density of localized electrons

$$s_{\mathrm{loc}}(t) = \frac{n_{\mathrm{loc}}(t)}{n_{\mathrm{max}}}. \tag{18}$$

Consequently, the localized-state oscillator vanishes when no electrons occupy localized states and gradually increases as trapping processes populate these states during laser excitation. The resonance frequency and damping constant are treated as constant parameters representing the optical response of localized defect states in amorphous fused silica. In contrast, the oscillator strength dynamically follows the localized electron density. To the best of our knowledge, no established literature

values exist for these parameters. The parameters are therefore introduced as phenomenological quantities motivated by the expected optical characteristics of localized defect-related states in amorphous fused silica. In particular, localized states are expected to exhibit lower transition energies than the valence-band resonance as well as stronger spectral broadening due to structural disorder and the distribution of defect environments. Accordingly, a lower resonance energy and an increased damping constant are employed compared to the valence-band oscillator.

Table 2. Parameters used for the localized-state Lorentz oscillator.

Parameter	Symbol	Value	Description
Resonance energy	$\hbar\omega_{0,\mathrm{loc}}$	3.0 eV	localized-state transition energy
Damping constant	$\hbar\Gamma_{\mathrm{loc}}$	1.5 eV	broad defect-state response
Oscillator strength	$\hbar\omega_{\mathrm{P,loc}}$	0.15 eV	phenomenological amplitude

S3.3 Drude response of free electrons

The optical response of free conduction-band electrons is described by the Drude model [12,14,15]

$$\varepsilon_{\mathrm{e}}(\omega,t) = -\frac{\omega_{\mathrm{p}}^2(t)}{\omega^2 + i\nu_{\mathrm{e}}\omega}, \tag{19}$$

where ν_{e} denotes the collision frequency of free electrons. The plasma frequency is determined by the free-electron density

$$\omega_{\mathrm{p}}(t) = \sqrt{\frac{n_{\mathrm{e}}(t)e^2}{\varepsilon_0 m_{\mathrm{eff}}}}. \tag{20}$$

The collision frequency is related to the electron collision time τ_{c} via

$$\nu_{\mathrm{e}} = \frac{1}{\tau_{\mathrm{c}}}.$$

The effective electron mass and electron collision time entering the Drude term are represented by effective literature-based quantities. The effective mass is set to $m_{\mathrm{eff}} = 0.5\,m_{\mathrm{e}}$, consistent with values commonly employed in models of laser-excited fused silica[8]. The collision time is set to $\tau_{\mathrm{c}} = 1$ fs, which lies within the femtosecond range reported for dense electronic excitation in fused silica[16,17]. However, the literature does not provide a unique collision time. Values inferred from transient optical measurements and from fits using different Drude-type descriptions vary substantially with excitation conditions, carrier density, electron energy, wavelength, and the specific optical model used for their extraction. The values of m_{eff} and τ_{c} were kept constant for all investigated pulse durations and fluences and were not adjusted to reproduce individual ablation depths or diameters. The collision time directly affects the real and imaginary parts of the Drude contribution and therefore influences the calculated refractive index, extinction coefficient, reflectivity, absorption coefficient, and resulting energy deposition. Nevertheless, agreement at the level of the final ablation geometry cannot uniquely determine τ_{c}, since changes in the collision time may interact with the electron-density evolution and with other effective parameters of the dielectric model. Similar final geometries obtained using different parameter combinations therefore do not necessarily imply identical transient optical responses.

Possible temporal, density-dependent, or energy-dependent variations of the collision time and effective mass are not resolved in the present framework. Their quantitative determination would require time-resolved measurements of the complex optical response under the same excitation conditions. The Drude term should therefore be understood as an effective description of the transient quasi-free-electron response rather than as a fully microscopic representation of all carrier-scattering processes.

Table 3. Parameters used in the Drude model.

Parameter	Symbol	Value	Description
Effective mass	m_{eff}	$0.5m_{\mathrm{e}}$	effective electron mass[8]
Collision time	τ_{c}	1 fs	electron collision time[16,17]

S3.4 Modified Clausius–Mossotti relation

The excitation of electrons from the valence band reduces the number of bound dipoles contributing to the dielectric background. To account for this effect, a modified Clausius–Mossotti relation is used, following the formulation introduced in the literature. The density of electrons remaining in the valence band is given by

$$N_{\mathrm{VB}}(t) = N_0 - n_{\mathrm{e}}(t) - n_{\mathrm{loc}}(t), \tag{21}$$

where N_0 denotes the initial valence electron density, while $n_{\mathrm{e}}(t)$ and $n_{\mathrm{loc}}(t)$ represent the densities of free and localized electrons, respectively.

The high-frequency dielectric constant is then expressed using the modified Clausius–Mossotti relation [18,19]

$$\varepsilon_\infty(t) = 1 + \frac{\beta_{\mathrm{CM}}\left[N_0 - n_{\mathrm{e}}(t) - n_{\mathrm{loc}}(t)\right]}{1 - \beta_{\mathrm{CM}}\left[N_0 - n_{\mathrm{e}}(t) - n_{\mathrm{loc}}(t)\right]}. \tag{22}$$

This formulation explicitly links the high-frequency bound-electron background to the evolving electron populations. As electrons are excited from the valence band into localized or free states, the density of polarizable bound valence-band electrons decreases, leading to a reduction of ε_∞(t). In the limit of complete depletion of the valence-band population, the bound-electron background contribution approaches $\varepsilon_\infty = 1$, corresponding to the disappearance of this dipole-polarization contribution. It is important to note that this limiting behavior applies only to the high-frequency background term ε_∞(t), not to the full dielectric function ($\varepsilon(\omega$,t)). The excited electrons remain part of the optical response through the localized-state Lorentz contribution and, in particular, through the Drude response of quasi-free conduction-band electrons. Therefore, under strong ionization, the total dielectric function is expected to become increasingly dominated by the Drude term. Depending on the transient free-electron density, the Drude contribution may dominate the real part of the total permittivity, resulting in near-zero or negative values, as expected for a dense plasma. In the absence of excitation, the model reproduces the equilibrium dielectric background of fused silica (ε_∞ = 1.49). This approach ensures consistency between the rate-equation model and the optical response model, as both are governed by the same evolving electron densities.

S3.5 Derivation of optical properties

The hybrid dielectric function introduced above provides direct access to the transient optical properties of the material. The complex dielectric function is written as

$$\varepsilon(\omega,t) = \varepsilon_1(\omega,t) + i\,\varepsilon_2(\omega,t), \tag{23}$$

where ε_1 and ε_2 denote the real and imaginary parts, respectively. The corresponding complex refractive index is given by

$$\tilde{n}(t) = n(t) + i\,k(t) = \sqrt{\varepsilon(\omega,t)}. \tag{24}$$

Separating real and imaginary parts yields

$$n(t) = \sqrt{\frac{|\varepsilon| + \varepsilon_1}{2}}, \qquad k(t) = \sqrt{\frac{|\varepsilon| - \varepsilon_1}{2}}, \tag{25}$$

with

$$|\varepsilon| = \sqrt{\varepsilon_1^2 + \varepsilon_2^2}. \tag{26}$$

The reflectivity at normal incidence is then obtained from the Fresnel equations as

$$R(t) = \frac{(n(t)-1)^2 + k^2(t)}{(n(t)+1)^2 + k^2(t)}. \tag{27}$$

The absorption coefficient follows as

$$\alpha(t) = \frac{4\pi\,k(t)}{\lambda}, \tag{28}$$

where λ denotes the laser wavelength. These quantities provide the direct connection between the time-dependent dielectric response and experimentally accessible optical observables. In particular, the transient evolution of the reflectivity and absorption coefficient determines the effective energy deposition during the laser pulse.

It should be noted that the optical properties derived in this section are evaluated within a zero-dimensional (0D) framework, i.e., at a fixed spatial position corresponding to the center of the laser spot. Consequently, all quantities are treated as time-dependent only. In a spatially resolved description, the reflectivity becomes a function of the lateral position and time, $R(x,t)$, since each lateral position experiences a different local fluence and therefore a different transient electronic response at the surface. In contrast, the absorption coefficient depends not only on time and lateral position x, but also on depth z, such that it becomes a local quantity $\alpha(x,z,t)$. In the present section, the optical response is therefore restricted to the local 0D case. The spatial extension of the model is realized using a multi-column approach based on the independent column approximation (ICA), where the laser-irradiated domain is discretized into an array of laterally independent one-dimensional columns.

In the spatially resolved implementation, the local absorption coefficient $\alpha(x,z,t)$ is used to determine the transient attenuation of the laser field and the corresponding depth-dependent absorbed power density. The effective optical penetration depth therefore evolves dynamically with the electronic excitation and is not prescribed by a constant Lambert–Beer absorption length. In this sense, the increasing attenuation associated with the transient plasma-like response is included in the present framework. However, the model does not contain a full wave-optical or paraxial propagation treatment. Diffraction, nonlinear self-focusing, plasma-induced defocusing, phase evolution, interference effects, transverse beam reshaping, and electromagnetic field redistribution within a strongly inhomogeneous plasma layer are not explicitly resolved. The optical response of an expanding ablation plume after fulfillment of the material-removal criterion is likewise outside the present model.

S3.6 Parameter uncertainty and identifiability

Several quantities entering the opto-electronic model are associated with literature-based or phenomenological uncertainties. These include the bandgap, avalanche ionization coefficient, trapping time, recombination coefficient, effective electron mass, Drude collision time, and the resonance, damping, and oscillator-strength parameters of the localized-state Lorentz contribution. Their effective values may depend on wavelength, pulse duration, intensity, carrier density, electron temperature, transient structural state, experimental observable, and the model used for their extraction. All parameter values used in the present study were kept fixed across the complete set of pulse durations and fluences and were not adjusted individually to reproduce the corresponding experimental ablation geometries.

The comparison with experimentally measured ablation profiles, depths, diameters, and thresholds therefore evaluates the integrated response of the coupled framework, but it does not uniquely identify each internal microscopic parameter. Partially compensating effects cannot be excluded. For example, different combinations of excitation, scattering, and relaxation parameters may produce similar final ablation geometries while predicting different transient electron densities, refractive indices, extinction coefficients, reflectivities, or energy-deposition profiles.

A systematic numerical sensitivity analysis would provide information about the mathematical response of the model to a selected parameter space but would not, by itself, resolve this physical non-uniqueness. Varying one parameter at a time would neglect correlations and compensating effects, whereas simultaneous variation would require assumptions regarding admissible ranges, distributions, and mutual correlations that are not sufficiently constrained by the available ex-situ data. A physically meaningful reduction of these uncertainties requires time- and spatially resolved measurements under identical excitation conditions. In particular, pump–probe reflectometry and ellipsometry below and close to the ablation threshold could constrain the transient reflectivity, refractive index, and extinction coefficient. Under ablative conditions, complementary interferometric measurements would be required to distinguish the response of the excited condensed material from that of expanding or detached material. Such combined experimental and numerical parameter-identifiability studies represent a subsequent development of the present framework.

S4 Two-temperature model

S4.1 Coupling and one-dimensional formulation

The two-temperature model (TTM), originally introduced to describe nonequilibrium energy exchange between electrons and lattice subsystems[20,21], is directly coupled to the opto-electronic model described in Section S3 through the depth- and time-resolved conduction-band electron density $n_\mathrm{e}(z,t)$ and the absorbed volumetric power density. The thermal response is described by a one-dimensional formulation resolving the depth-dependent evolution of the electron temperature $T_\mathrm{e}(z,t)$ and the lattice temperature $T_\mathrm{l}(z,t)$. The coupled energy balance equations read

$$C_\mathrm{e}\frac{\partial T_\mathrm{e}}{\partial t} = \frac{\partial}{\partial z}\left(k_\mathrm{e}\frac{\partial T_\mathrm{e}}{\partial z}\right) - G\left(T_\mathrm{e} - T_\mathrm{l}\right) + S(z,t), \tag{29}$$

$$C_\mathrm{l}\frac{\partial T_\mathrm{l}}{\partial t} = \frac{\partial}{\partial z}\left(k_\mathrm{l}\frac{\partial T_\mathrm{l}}{\partial z}\right) + G\left(T_\mathrm{e} - T_\mathrm{l}\right). \tag{30}$$

Here, C_e and C_l denote the volumetric heat capacities, k_e and k_l the thermal conductivities, and G the electron–phonon coupling factor. The source term $S(z,t)$ is obtained from the opto-electronic model and represents the locally absorbed volumetric power density. In contrast to conventional approaches based on Lambert–Beer absorption, $S(z,t)$ is not described by a predefined exponential decay. Instead, it results from the transient optical response of the material, leading to a depth- and time-dependent energy deposition. Only conduction-band electrons are considered as part of the thermal electron subsystem. Trapped carriers, although included in the opto-electronic model to describe the transient dielectric response, do not contribute to the electronic heat capacity or to the electron–phonon coupling.

S4.2 Analytical description of the electron subsystem

The electron subsystem is described by the volumetric electron heat capacity C_e, the electron–phonon coupling factor G, and the electron thermal conductivity k_e. In the present model, these quantities are linked to the transient conduction-band electron density $n_\mathrm{e}(z,t)$ obtained from the opto-electronic model.

The volumetric electron heat capacity is described using the analytical interpolation reported by Tsaturyan et al.[6] based on density-dependent DFT calculations:

$$C_\mathrm{e}(n_\mathrm{e}) = 25.4 - 22.8\exp\left(-\frac{n_\mathrm{e}}{5.1\times 10^{23}}\right) - 2.5\exp\left(-\frac{n_\mathrm{e}}{3.5\times 10^{21}}\right), \tag{31}$$

where n_e is given in $\mathrm{cm^{-3}}$ and C_e in $\mathrm{J\,cm^{-3}\,K^{-1}}$. In the numerical implementation, Eq. (31) is converted to SI units.

The electron–phonon coupling factor is described by an analytical fit constructed in the present work based on the conduction-band data reported by Tsaturyan et al.[6], for which no explicit analytical expression is provided, and is given by

$$G(n_\mathrm{e}) = 6.2\times 10^{12} + 1.0\times 10^{11}\left[1-\exp\left(-\left(\frac{n_\mathrm{e}}{1.6\times 10^{19}}\right)^{0.36}\right)\right] + 5.0\times 10^{18}\left[1-\exp\left(-\left(\frac{n_\mathrm{e}}{8.3\times 10^{21}}\right)^{0.91}\right)\right], \tag{32}$$

where G is given in $\mathrm{W\,m^{-3}\,K^{-1}}$ and n_e in $\mathrm{cm^{-3}}$. Both functions are visualized in Fig. 2 for reference. The restriction to the conduction-band contribution is applied consistently with the present two-temperature formulation. In the underlying reference, separate coupling contributions are reported for valence-band and conduction-band electrons. A simultaneous thermodynamic treatment of both subsystems would require an extended multi-temperature description. In the present model, only conduction-band electrons are therefore included in the thermal electron subsystem, while trapped electrons are accounted for in the opto-electronic model but are not introduced as an additional thermal subsystem. In contrast to the effective trapping and recombination times employed in the rate-equation model, electron–phonon relaxation is not represented by a single prescribed relaxation time. Its characteristic timescale is determined locally by the electron heat capacity and electron–phonon coupling factor, both of which vary with the transient conduction-band electron density. Consequently, the electron–lattice energy-transfer dynamics evolve with the excitation level. This density-dependent treatment is a central distinction from classical two-temperature formulations employing constant electron heat capacities, coupling factors, or phenomenological relaxation times.

The electron thermal conductivity k_e is described in a Drude-type framework as an effective transport quantity of the excited electron subsystem[22–24]. In this formulation, k_e is related to the electron heat capacity, a characteristic electron velocity, and an effective relaxation time. Accordingly, k_e is treated as a transient material property within the laser-excited region and enters the diffusion term of Eq. (29).

S4.3 Analytical description of the lattice subsystem

The lattice subsystem is described by the lattice temperature $T_\mathrm{l}(z,t)$ and accounts for heat diffusion, energy exchange with the excited electron subsystem, melting, and thermally induced material removal. In the present model, the lattice-specific heat capacity is assumed to be constant,

$$c_\mathrm{l} = 730\,\mathrm{J\,kg^{-1}\,K^{-1}}, \tag{33}$$

with a mass density of

$$\rho = 2200\,\mathrm{kg\,m^{-3}}. \tag{34}$$

Accordingly, the baseline volumetric lattice heat capacity is given by

$$C_{\mathrm{l},0} = \rho c_\mathrm{l}. \tag{35}$$

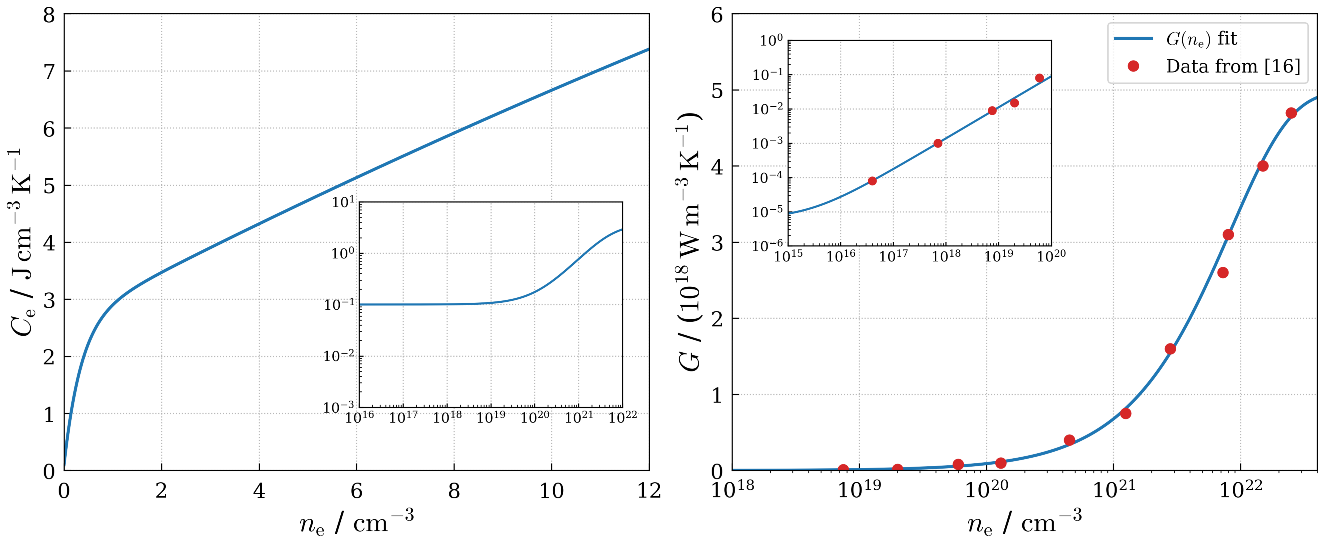

Figure 2. Density-dependent material functions of the electron subsystem. **Left:** Volumetric electron heat capacity C_e as a function of the conduction-band electron density n_e using the analytical interpolation adopted from Tsaturyan et al.[6]. The inset shows the same dependence on a logarithmic scale. **Right:** Electron–phonon coupling factor G as a function of the conduction-band electron density n_e. The solid line represents the analytical fit constructed in the present work based on the conduction-band data reported by Tsaturyan et al.[6]. The red markers indicate the corresponding reference data points. The inset shows the low-density regime on a logarithmic scale.

The lattice thermal conductivity[25] is likewise treated as constant,

$$k_l = 1.4\ \mathrm{W\,m^{-1}\,K^{-1}}. \tag{36}$$

To account for melting, the latent heat of fusion is introduced through an effective volumetric lattice heat capacity using a mushy-zone formulation[26–28]. The melting temperature[29] is set to

$$T_m = 1988\ \mathrm{K}, \tag{37}$$

and the latent heat of fusion[29] is

$$L_{fus} = 1.56 \times 10^5\ \mathrm{J\,kg^{-1}}. \tag{38}$$

Instead of a discontinuous phase transition at T_m, the latent heat is distributed over a finite temperature interval[26,30] of width

$$\Delta T_{melt} = 100\ \mathrm{K}, \tag{39}$$

centered around T_m. With

$$T_1 = T_m - \frac{\Delta T_{melt}}{2}, \qquad T_2 = T_m + \frac{\Delta T_{melt}}{2}, \tag{40}$$

the effective volumetric lattice heat capacity is written as

$$C_l(T_l) = \begin{cases} \rho c_l, & T_l < T_1, \\ \rho c_l + \rho \dfrac{L_{fus}}{\Delta T_{melt}}, & T_1 \le T_l \le T_2, \\ \rho c_l, & T_l > T_2. \end{cases} \tag{41}$$

This approach ensures a numerically smooth treatment of the solid-to-liquid transition while preserving the total latent heat contribution. For diagnostics and threshold evaluation, the lattice enthalpy is additionally defined as

$$H_l(T_l) = \rho c_l (T_l - T_0) + \rho L_{fus}\, \phi(T_l), \tag{42}$$

where $T_0 = 300$ K is the initial temperature and $\phi(T_l)$ denotes the phase fraction,

$$\phi(T_l) = \begin{cases} 0, & T_l < T_1, \\ \dfrac{T_l - T_1}{T_2 - T_1}, & T_1 \leq T_l \leq T_2, \\ 1, & T_l > T_2. \end{cases} \tag{43}$$

Thermally induced material removal is described by a temperature-based phase-explosion surrogate criterion. In laser-excited condensed matter, phase explosion is commonly associated with the rapid decomposition of a metastable superheated liquid when approaching the thermodynamic critical point. Experimental and theoretical studies have shown that this transition typically occurs below the critical temperature, within a range of approximately 0.8–$0.9\,T_c$, due to the loss of mechanical stability in the superheated liquid phase[31–33]. For silica, the thermodynamic critical temperature was taken as

$$T_c = 4862 \text{ K}, \tag{44}$$

following the literature value obtained from a semi-empirical wide-range equation-of-state description[29,34,35]. The temperature threshold for material removal is then defined as

$$T_{PE} = f_{PE}\, T_c, \tag{45}$$

with

$$f_{PE} = 0.90, \tag{46}$$

which yields

$$T_{PE} \approx 4.38 \cdot 10^3 \text{ K}. \tag{47}$$

In the numerical implementation, a lattice cell is treated as removed once

$$T_l \geq T_{PE}. \tag{48}$$

After removal, the corresponding cell is excluded from subsequent source deposition, electron–phonon coupling, and heat conduction, thereby representing the local loss of condensed material.

S5 Numerical implementation and simulation workflow

S5.1 Temporal and spatial discretization

The numerical implementation is based on a transient one-dimensional discretization along the depth coordinate z. The optical-electronic model provides the absorbed volumetric power density $p_{abs}(z,t)$ and the free-electron density $n_e(z,t)$ on a common depth-time grid, which is used as the input for the subsequent thermal calculation.

The spatial domain is discretized from the material surface ($z = 0$) into the bulk using a uniform depth grid. The temporal evolution is resolved on the time grid obtained from the optical-electronic model, such that the source term and the free-electron density remain consistently aligned in both space and time. For the thermal solver, the coordinate axes are internally converted to SI units, while the imported source and carrier-density fields are preserved on the original numerical grid.

The electron temperature $T_e(z,t)$ and the lattice temperature $T_l(z,t)$ are initialized with the ambient temperature T_0. The subsequent temporal evolution is then evaluated sequentially for each time increment over the full simulation window.

S5.2 Coupling strategy and time integration

The optical–electronic model and the two-temperature model are coupled through the transient absorbed volumetric power density $P_{abs}(z,t)$ and the free-electron density $n_e(z,t)$, which are evaluated consistently for each depth and time increment. These quantities serve as the local source term and as input for the material parameters of the thermal subsystem.

The temporal evolution of the electron and lattice temperatures is obtained by sequential time stepping over the prescribed simulation window. For each time increment, the local source term is assigned from the precomputed field $P_{abs}(z,t)$, while the corresponding free-electron density $n_e(z,t)$ is used to evaluate the density-dependent quantities entering the two-temperature equations.

The coupled energy balance equations are integrated using an explicit time integration scheme. To ensure numerical stability in the presence of steep temporal gradients, each global time step is subdivided into smaller substeps, and the solution is

advanced iteratively within each interval. All material parameters and coupling terms are updated locally within these substeps based on the instantaneous state variables.

Material removal is accounted for during the time integration by evaluating a temperature-based criterion. Once the lattice temperature in a given depth cell exceeds the prescribed threshold, the corresponding cell is excluded from further energy deposition and coupling processes. This is implemented by suppressing the local source term and transport contributions in the affected region for all subsequent time steps.

This procedure yields a fully transient solution of the coupled system in which the optical excitation and thermal response remain consistently linked through the local source and carrier-density fields.

S5.3 Numerical solution of the two-temperature model

The spatial derivatives in the two-temperature equations are discretized using finite differences on the uniform depth grid. The diffusive heat transport in both the electron and lattice subsystems is evaluated through second-order spatial derivatives, yielding a local update of the temperature fields based on the neighboring grid points.

Zero-flux boundary conditions are applied at the surface and at the maximum simulation depth, corresponding to vanishing temperature gradients at both boundaries. This ensures that no artificial energy flux enters or leaves the computational domain during the simulation.

All temperature-dependent and density-dependent quantities entering the two-temperature equations are evaluated locally at each depth and time increment. In particular, the electron heat capacity, the electron–phonon coupling factor, and the electron thermal conductivity are updated within each time step based on the instantaneous values of the state variables.

The numerical update of the temperature fields is performed sequentially for the electron and lattice subsystems within each substep of the time integration. The diffusion terms, coupling terms, and source terms are consistently evaluated using the current state of the system, resulting in a fully explicit update scheme for both temperature fields.

S5.4 Phase change and material removal

Phase transitions are incorporated in the numerical scheme through the effective heat capacity formulation described in Section S4.3. Within the melting interval, the latent heat contribution is included locally via the temperature-dependent lattice heat capacity, ensuring a continuous transition between solid and liquid phases without introducing discontinuities in the temperature field.

Thermally induced material removal is implemented by evaluating the lattice temperature against the prescribed phase-explosion threshold. Once the local lattice temperature exceeds this threshold, the corresponding depth cell is marked as removed and excluded from further contributions to the energy balance.

In the numerical implementation, this removal is enforced by suppressing the local source term as well as all coupling and transport contributions in the affected cells for all subsequent time steps. As a result, removed regions no longer participate in energy absorption, electron–phonon coupling, or heat conduction.

The position of the effective material surface is updated dynamically during the simulation by identifying the first non-removed cell along the depth coordinate. All diagnostic quantities related to the surface are evaluated with respect to this moving boundary, enabling a consistent description of the evolving material interface without remeshing of the computational grid.

S5.5 Quasi-two-dimensional extension

The extension from the one-dimensional depth-resolved model to a quasi-two-dimensional description is achieved by applying an independent column approximation (ICA) along the lateral coordinate x. The incident laser fluence is distributed laterally according to the Gaussian spatial beam profile, and each lateral position x_i is treated as an independent one-dimensional column. The lateral domain is discretized with a column width of ($\Delta x = 200\,\mathrm{nm}$). For each column, the optical–electronic model and the two-temperature model are evaluated using the locally assigned fluence $H(x_i)$. This results in a set of independent depth-resolved solutions $T_\mathrm{e}(z,t;x_i)$, $T_\mathrm{l}(z,t;x_i)$, and corresponding material modification quantities for each lateral position. The global response of the system is then reconstructed by assembling these individual solutions along the lateral coordinate x. This approach enables the computation of spatially resolved quantities such as lateral reflectivity distributions, melt depths, ablation depths, and resulting cross-sectional ablation profiles. The ICA assumes that lateral energy transport between neighboring columns is negligible on the timescales considered. Consequently, lateral heat diffusion and hydrodynamic coupling of the melt layer are not explicitly resolved. Under this assumption, the computational complexity is significantly reduced while retaining the essential spatial variation induced by the lateral fluence distribution.

S6 Representative model outputs

The following section provides representative simulation results to illustrate the behavior of the coupled model. A detailed analysis and comparison with experimental data is presented in the main manuscript. Representative simulations are shown for a pulse duration of 200 fs and a fluence of 15 J cm^{-2}.

S6.1 Optical–electronic response

The coupled behavior of the electron excitation dynamics and the transient optical response is illustrated for the representative simulation conditions (Figure 3). The results reflect the direct linkage between the rate-equation-based electron density evolution (Section S2) and the hybrid dielectric response model (Section S3). The left panel visualizes the temporal buildup of the conduction-band electron density resulting from the individual source and loss terms of the rate equation. The right panel shows the corresponding evolution of the optical properties, which are directly determined by the instantaneous electron density through the hybrid dielectric response. Together, both representations illustrate the causal connection between electronic excitation and transient optical behavior within the model framework. All quantities shown correspond to the central column ($x = 0$), i.e., the peak fluence of 15 J cm^{-2}.

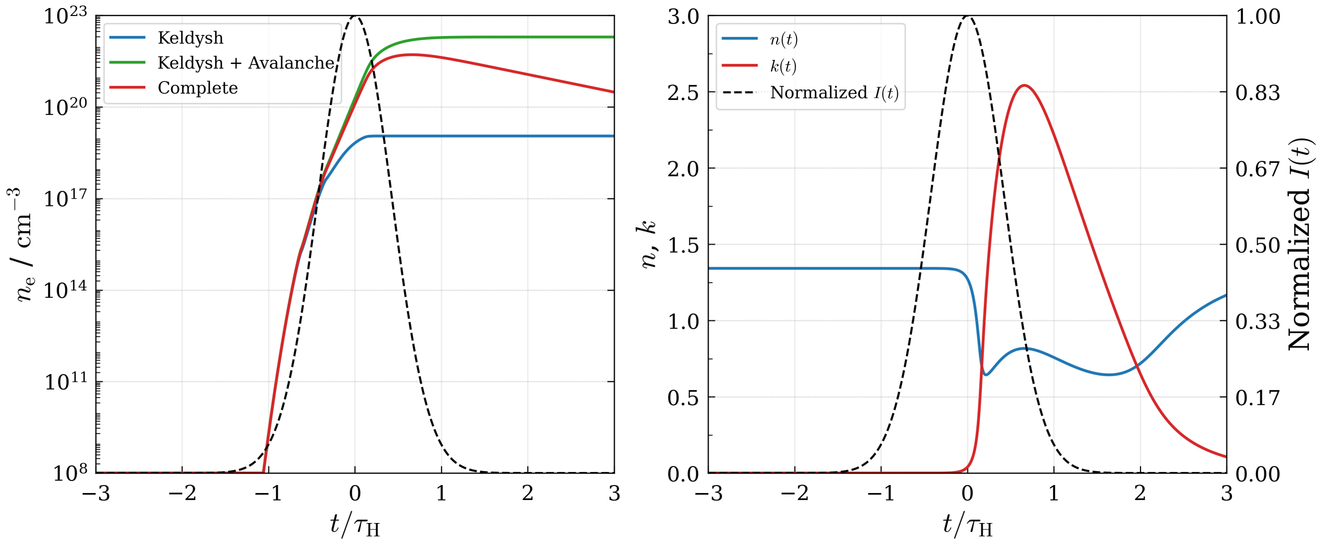

Figure 3. Time-resolved surface response illustrating the coupling between electron excitation dynamics and the transient optical properties. **Left:** Evolution of the conduction-band electron density $n_e(t)$ showing the contributions of the individual terms of the rate equation (Eq. 8), including photoionization, avalanche ionization, and loss mechanisms. **Right:** Corresponding refractive index $n(t)$ and extinction coefficient $k(t)$ obtained from the hybrid dielectric response model. The dashed line indicates the normalized laser intensity.

S6.2 Energy deposition and spatial distribution

The spatial distribution of the reflected and absorbed energy is illustrated in Figure 4 based on the transient optical response described in Section S3. The lateral reflectivity distribution is obtained from the independent column approximation (ICA), while the depth-resolved quantities correspond to the central column ($x = 0$). The reflectivity determines the fraction of incident energy entering the material, while the absorption coefficient governs its depth-dependent attenuation. The resulting absorbed volumetric power density $P_{abs}(z,t)$ represents the local energy deposition within the material and corresponds to the source term $S(z,t)$ used in the two-temperature model (Eq. 29).

S6.3 Thermal response and material modification

The thermal response of the material is determined by the absorbed volumetric power density $S(z,t)$ introduced in Section S6.2 and evaluated within the two-temperature framework described in Section S4. Figure 5 shows the resulting evolution of the lattice subsystem for the central column ($x = 0$), where the left panel reflects the implementation of the phase-transition model through the effective volumetric heat capacity, with the mushy zone appearing as a finite temperature interval centered around the melting temperature, while regions removed during the simulation are excluded from further thermal evolution and therefore do not contribute to the heat capacity. The corresponding lattice temperature distribution in the right panel governs the phase

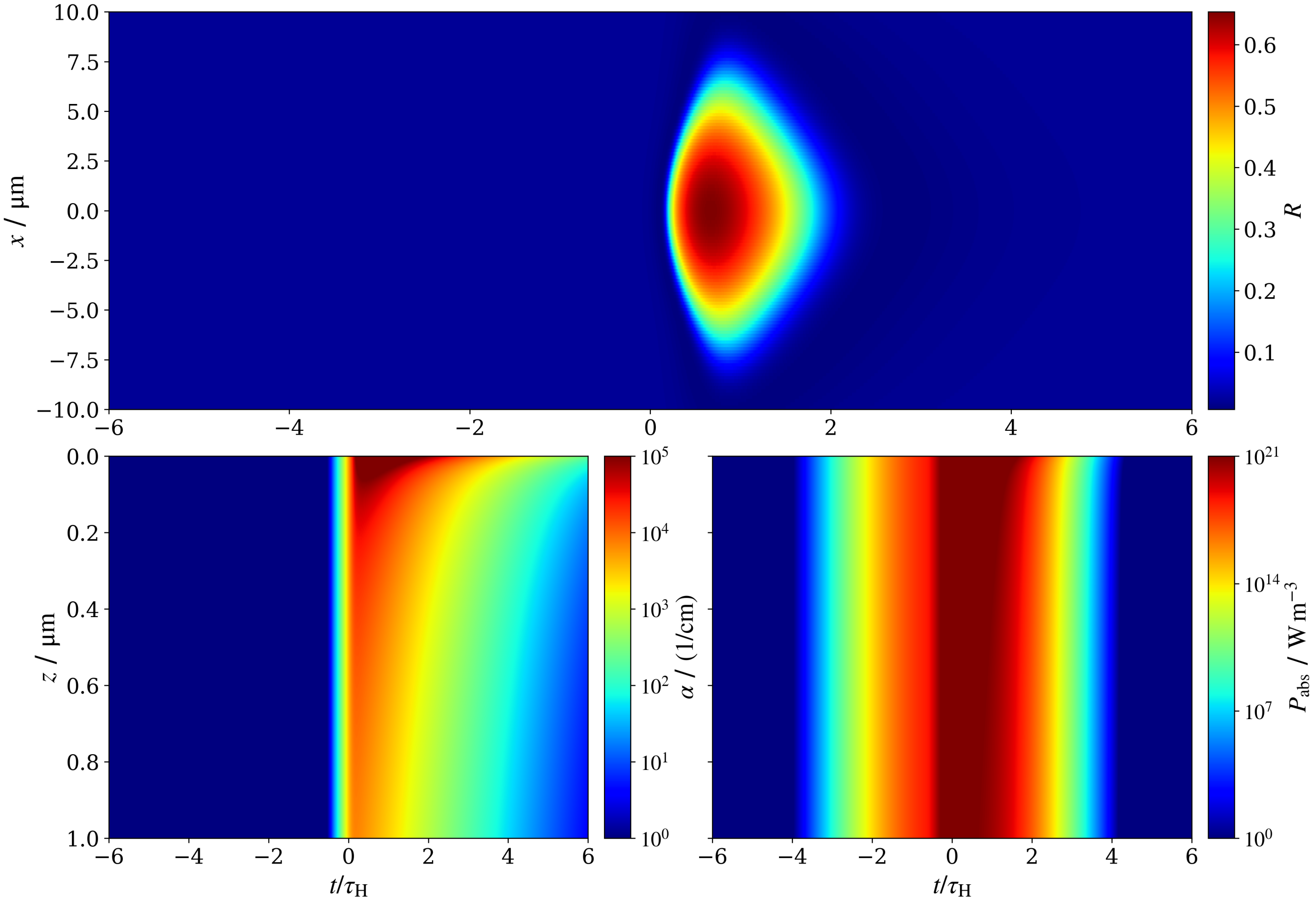

Figure 4. Spatial and temporal distribution of the laser energy within the model framework. **Top:** Lateral and time-resolved reflectivity $R(x,t)$ reconstructed using the independent column approximation. **Bottom left:** Depth- and time-dependent absorption coefficient $\alpha(z,t)$ for the central column ($x=0$). **Bottom right:** Corresponding absorbed volumetric power density $P_{abs}(z,t)$.

state of the material, where the melting isotherm (T_m) marks the onset of the phase transition and the higher temperature threshold (T_{PE}) defines the onset of material removal, such that the temperature field directly determines both the transient melt region and the final ablation depth. Figure 6 shows the resulting spatial material modification obtained from the full model, where the independently evaluated columns are combined using the independent column approximation, yielding a two-dimensional representation of the ablation structure in the top view and the corresponding lateral depth profile in the cross-section. The surface is defined at $z=0$, with negative values indicating material removal, while the color scale in the top view represents the magnitude of the ablation depth, providing a compact representation of the transition from energy deposition to permanent material modification.

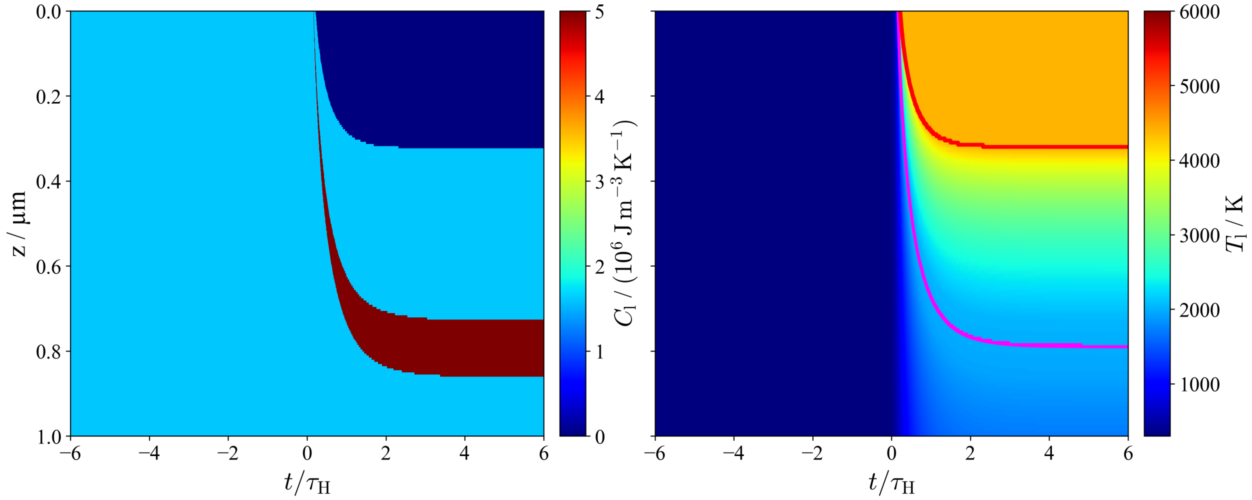

Figure 5. Thermal response of the lattice subsystem for the central column ($x = 0$). **Left:** Effective volumetric lattice heat capacity $C_l(z,t)$, illustrating the mushy-zone formulation used to model the phase transition. **Right:** Lattice temperature $T_l(z,t)$. The magenta line indicates the melting isotherm ($T_m = 1988$ K), while the red line marks the temperature threshold used for material removal (T_{PE}).

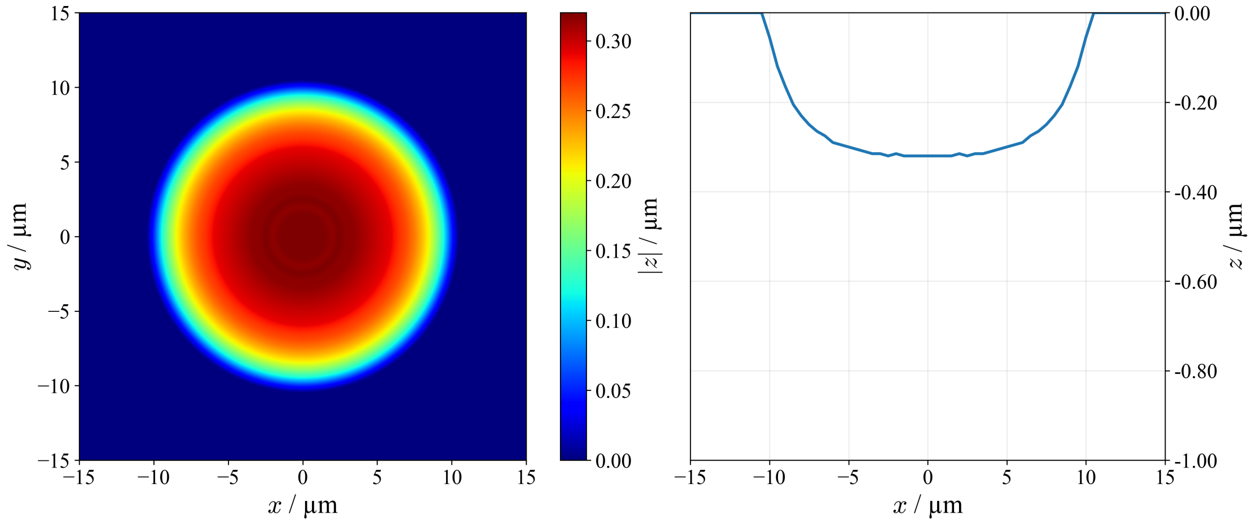

Figure 6. Spatial representation of the resulting ablation structure obtained from the two-temperature model. **Left:** Top view of the ablation profile reconstructed using the independent column approximation. The color scale in the top view represents the magnitude of the ablation depth. **Right:** Corresponding cross-sectional depth profile of the ablation depth $z(x)$. The surface is defined at $z = 0$, and negative values indicate material removal.

References

1. Keldysh, L. V. Ionization in the field of a strong electromagnetic wave. *Sov. Phys. JETP* **20**, 1307–1314 (1965).
2. Stuart, B. C., Feit, M. D., Rubenchik, A. M., Shore, B. W. & Perry, M. D. Laser-induced damage in dielectrics with nanosecond to subpicosecond pulses. *Phys. Rev. Lett.* **74**, 2248–2251, DOI: 10.1103/PhysRevLett.74.2248 (1995).
3. Rethfeld, B. Unified model for the free-electron avalanche in laser-irradiated dielectrics. *Phys. Rev. Lett.* **92**, 187401, DOI: 10.1103/PhysRevLett.92.187401 (2004).
4. Balling, P. & Schou, J. Femtosecond-laser ablation dynamics of dielectrics: basics and applications for thin films. *Reports on Prog. Phys.* **76**, 036502, DOI: 10.1088/0034-4885/76/3/036502 (2013).
5. Tsaturyan, A., Kachan, E., Stoian, R. & Colombier, J.-P. Ultrafast bandgap narrowing and cohesion loss of photoexcited fused silica. *The J. Chem. Phys.* **156**, DOI: 10.1063/5.0096530 (2022).
6. Tsaturyan, A., Kachan, E., Stoian, R. & Colombier, J.-P. Unraveling the electronic properties in sio2 under ultrafast laser irradiation. *npj Comput. Mater.* **10**, 200, DOI: 10.1038/s41524-024-01350-2 (2024).
7. Tsaturyan, A. A., Kachan, E., Stoian, R. & Colombier, J.-P. Excited-state dynamics and optical properties of silica under ultrafast laser irradiation. *Adv. Phys. Res.* **4**, 2400106, DOI: 10.1002/apxr.202400106 (2025).
8. Pflug, T., Olbrich, M. & Horn, A. Electron dynamics in fused silica after strong field laser excitation detected by spectroscopic imaging pump-probe ellipsometry. *Phys. Rev. B* **106**, 014307, DOI: 10.1103/PhysRevB.106.014307 (2022).
9. Jiang, L. & Tsai, H.-L. Plasma modeling for ultrashort pulse laser ablation of dielectrics. *J. Appl. Phys.* **100**, DOI: 10.1063/1.2216882 (2006).
10. Lenzner, M. *et al.* Femtosecond optical breakdown in dielectrics. *Phys. review letters* **80**, 4076, DOI: 10.1103/PhysRevLett.80.4076 (1998).
11. Kaiser, A., Rethfeld, B., Vicanek, M. & Simon, G. Microscopic processes in dielectrics under irradiation by subpicosecond laser pulses. *Phys. review B* **61**, 11437, DOI: 10.1103/PhysRevB.61.11437 (2000).
12. Fujiwara, H. *Spectroscopic Ellipsometry: Principles and Applications* (John Wiley & Sons, 2007).
13. Rakić, A. D., Djurišić, A. B., Elazar, J. M. & Majewski, M. L. Optical properties of metallic films for vertical-cavity optoelectronic devices. *Appl. Opt.* **37**, 5271, DOI: 10.1364/AO.37.005271 (1998).
14. Kittel, C. *Introduction to Solid State Physics* (Wiley, 2011), 8 edn.
15. Ashcroft, N. W. & Mermin, N. D. *Solid State Physics* (Cengage Learning, 2020).
16. Wu, A. Q., Chowdhury, I. H. & Xu, X. Femtosecond laser absorption in fused silica: Numerical and experimental investigation. *Phys. Rev. B* **72**, 085128, DOI: 10.1103/PhysRevB.72.085128 (2005).
17. Sun, Q. *et al.* Measurement of the collision time of dense electronic plasma induced by a femtosecond laser in fused silica. *Opt. Lett.* **30**, 320–322, DOI: 10.1364/OL.30.000320 (2005).
18. Guizard, S. *et al.* Femtosecond laser ablation of transparent dielectrics: measurement and modelisation of crater profiles. *Appl. Surf. Sci.* **186**, 364–368, DOI: 10.1016/S0169-4332(01)00681-X (2002).
19. Winkler, T. T. *Laser amplification in excited dielectrics* (Universitätsbibliothek Kassel, 2018).
20. Anisimov, S., Kapeliovich, B., Perelman, T. *et al.* Electron emission from metal surfaces exposed to ultrashort laser pulses. *Zh. Eksp. Teor. Fiz* **66**, 375–377 (1974).
21. Fujimoto, J. G., Liu, J. M., Ippen, E. P. & Bloembergen, N. Femtosecond laser interaction with metallic tungsten and nonequilibrium electron and lattice temperatures. *Phys. Rev. Lett.* **53**, 1837–1840, DOI: 10.1103/PhysRevLett.53.1837 (1984).
22. Ashcroft, N. W. & Mermin, N. D. *Solid State Physics* (Brooks Cole, 1976), 1 edn.
23. Jiang, L. & Tsai, H.-L. A plasma model combined with an improved two-temperature equation for ultrafast laser ablation of dielectrics. *J. Appl. Phys.* **104**, DOI: 10.1063/1.3006129 (2008).
24. Lin, Z., Zhigilei, L. V. & Celli, V. Electron-phonon coupling and electron heat capacity of metals under conditions of strong electron-phonon nonequilibrium. *Phys. Rev. B* **77**, 075133, DOI: 10.1103/PhysRevB.77.075133 (2008).
25. Combis, P., Rullier, J.-L., Courtois, M. & Lescoute, E. Evaluation of the fused silica thermal conductivity at high temperature. *J. Appl. Phys.* **112**, 093512, DOI: 10.1063/1.4764904 (2012).

26. Voller, V. R., Cross, M. & Markatos, N. C. An enthalpy method for convection/diffusion phase change. *Int. J. for Numer. Methods Eng.* **24**, 271–284, DOI: 10.1002/nme.1620240119 (1987).

27. Brent, A. D., Voller, V. R. & Reid, K. J. Enthalpy-porosity technique for modeling convection-diffusion phase change: Application to the melting of a pure metal. *Numer. Heat Transf.* **13**, 297–318, DOI: 10.1080/10407788808913615 (1988).

28. Voller, V. R. & Swaminathan, C. R. General source-based method for solidification phase change. *Numer. Heat Transf.* **19**, 175–189 (1991).

29. Iosilevskiy, I., Gryaznov, V. & Solov'ev, A. Properties of high-temperature phase diagram and critical point parameters in silica. *arXiv preprint arXiv:1312.7592* DOI: 10.48550/arXiv.1312.7592 (2013).

30. Tayler, A. Free and moving boundary problems. by j. crank. clarendon, oxford, 1984. 425 pp.£ 45.00. *J. Fluid Mech.* **158**, 532–533, DOI: 10.1017/S0022112085212750 (1985).

31. Miotello, A. & Kelly, R. Laser-induced phase explosion: new physical problems when a condensed phase approaches the thermodynamic critical temperature. *Appl. Phys. A* **69**, S67–S73, DOI: 10.1007/s003399900296 (1999).

32. Porneala, C. & Willis, D. A. Observation of nanosecond laser-induced phase explosion in aluminum. *Appl. Phys. Lett.* **89**, 211121, DOI: 10.1063/1.2393158 (2006).

33. Porneala, C. & Willis, D. A. Effect of the dielectric transition on laser-induced phase explosion in metals. *Int. J. Heat Mass Transf.* **49**, 1928–1936, DOI: 10.1016/j.ijheatmasstransfer.2005.11.005 (2006).

34. Faik, S., Tauschwitz, A., Maruhn, J. & Iosilevskiy, I. Lifetime of metastable states in ion-beam irradiated sio_2 foils. In Fortov, V. (ed.) *Physics of Extreme States of Matter*, 105 (IPCP RAS, Chernogolovka, 2011).

35. Faik, S., Tauschwitz, A., Maruhn, J. & Iosilevskiy, I. Mpqeos-jwgu: A new equation-of-state package for warm/hot dense matter. GSI Annual Report, GSI Helmholtzzentrum für Schwerionenforschung (2011).
